# Function-preserving photooxidation of AS1411 aptamer by a G-quadruplex-guided photosensitizer

**DOI:** 10.1039/d6sc06144c

**Published:** 2026-10-05

**Authors:** Maria V. Cottini, Karolina Saczuk, Jakub Trojnar, Lucía López-Pacios, Dario Puchán Sánchez, Arthur H. G. David, Ikenna Obi, Nasim Sabouri, Juan J. Nogueira, Marta Dudek, Clément Cabanetos, Lara Martínez-Fernández, Jan Jamroskovic, Marco Deiana

**Affiliations:** a Department of Microbial Genetics, Slovak Academy of Sciences, Institute of Molecular Biology Dubravska cesta 21 845 51 Bratislava Slovakia jan.jamroskovic@savba.sk; b Institute of Advanced Materials, Faculty of Chemistry, Wrocław University of Science and Technology Wyb. Wyspiańskiego 27 Wrocław 50-370 Poland m.deiana@pwr.edu.pl; c Department of Chemistry, Universidad Autónoma de Madrid Calle Francisco Tomás y Valiente, 7 28049 Madrid Spain; d Univ Angers, CNRS MOLTECH-ANJOU SFR MATRIX F-49000 Angers France; e Department of Medical Biochemistry and Biophysics, Umeå University Umeå 90187 Sweden; f Science for Life Laboratory, Umeå University Umeå Sweden; g IADCHEM, Institute for Advanced Research in Chemistry, Universidad Autónoma de Madrid Calle Francisco Tomás y Valiente, 7 28049 Madrid Spain; h Departamento de Química Física de Materiales, Instituto de Química Física Blas Cabrera, CSIC Madrid 28006 Spain lmartinez@iqf.csic.es

## Abstract

AS1411 is a G-quadruplex (G4) aptamer that binds nucleolin (NCL) and is widely used to promote cancer-associated delivery of photoactive cargos. Yet the fate of AS1411 after photosensitizer loading and light activation remains unclear as reactive oxygen species (ROS) generation could disrupt its guanine-rich fold and abolish NCL recognition, or remain locally confined while preserving targeting function. Here, we address this question using DBI-POE, a heavy-atom-free dibenzothioxanthene imide photosensitizer combining G4 recognition, fluorescence activation, and ROS generation. In this system, AS1411 converts weakly emissive, radical-prone DBI-POE aggregates into a fluorescent G4-bound complex with singlet oxygen (^1^O_2_)-biased reactivity and attenuated radical-probe responses. ^1^H NMR spectroscopy, quantum-mechanical (QM) calculations, molecular dynamics (MD) simulations, and primer-extension mapping support preferential DBI-POE association with the 3′-terminal tetrad region and are consistent with a partially intercalated binding model. Upon irradiation, this complex undergoes site-localized guanine oxidation. This damage is suppressed under non-G4-forming conditions and in G4-disrupted controls. Remarkably, oxidation preserves the BG4-recognized G4 epitope and full-length NCL binding, defining a DBI-POE-specific, function-preserving mode of aptamer-guided photosensitization.

## Abstract 2

AS1411 reprograms DBI-POE photochemistry toward site-localized guanine oxidation while preserving G4 structure and nucleolin recognition, revealing a function-preserving mode of aptamer-guided photosensitization.

## Graphical abstract

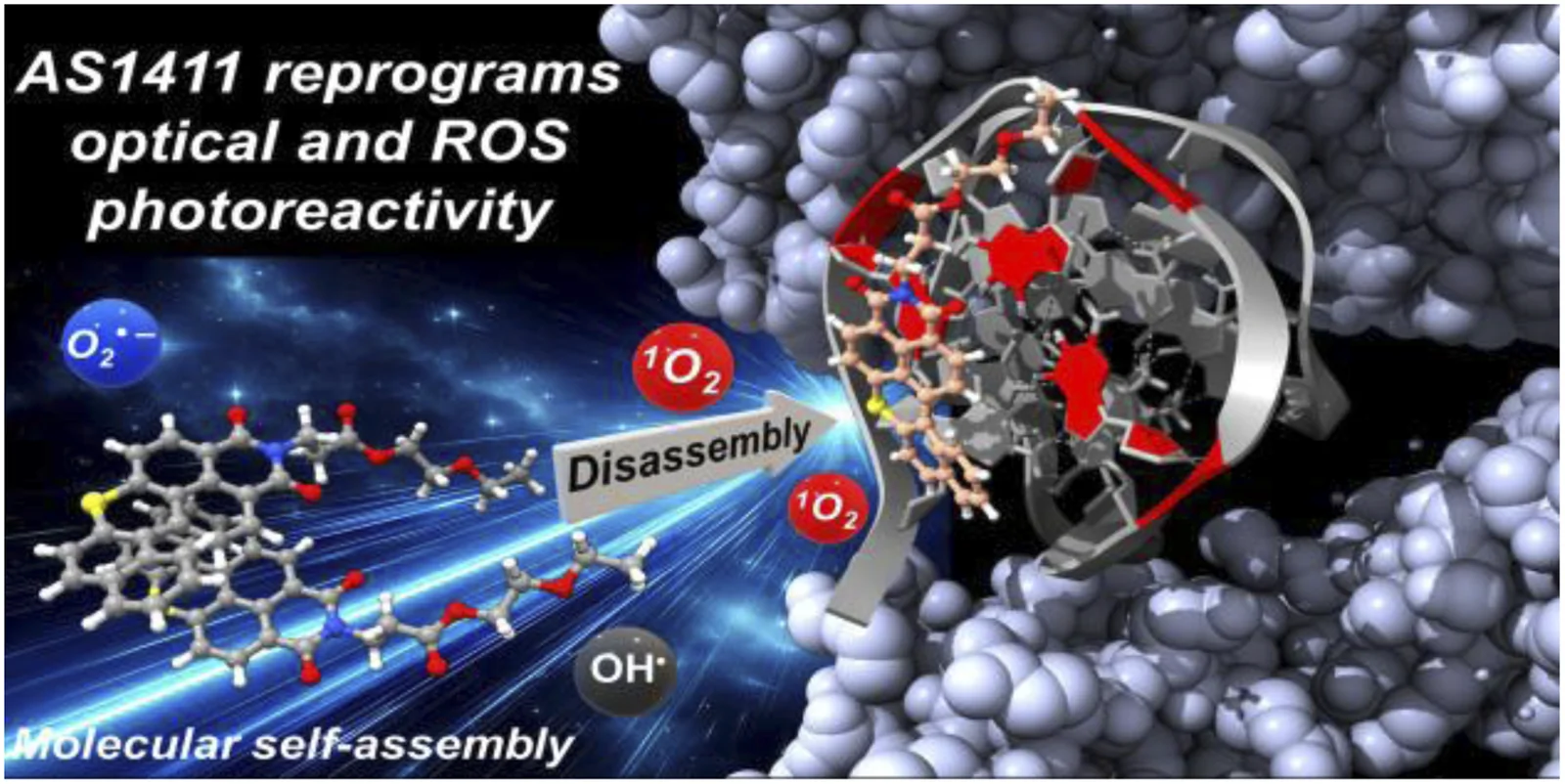

## Introduction

G-quadruplexes (G4s) are noncanonical nucleic acid structures formed by stacked guanine tetrads and stabilized by monovalent cations.^1,2^ They are widely recognized as regulatory elements in genome biology, where their formation has been linked to transcription, replication, genome stability, and DNA-damage responses.^3–7^ Yet G4s are not only structural motifs. Their stacked guanine quartets create extended, electron-rich π-surfaces that combine high ligand affinity with intrinsic susceptibility to oxidation.^8–11^ Because guanine is the most readily oxidized canonical nucleobase, folded G4s provide chemically privileged environments in which excited-state processes, charge transfer, and oxidative damage can be organized at the molecular scale.^9,12–15^

This chemical dimension creates an opportunity that is not captured by conventional descriptions of G4 ligand binding. Most G4-targeted systems are judged by affinity, selectivity, and thermal stabilization, but these parameters do not necessarily predict whether binding can be converted into a controlled chemical outcome.^16^ Photoactive ligands offer a more powerful possibility: molecular recognition can be coupled directly to light-triggered reactivity.^17–25^ Upon irradiation, a DNA-bound photosensitizer can populate an excited triplet state that initiates oxidative damage through two principal photochemical pathways. In Type I photosensitization, the triplet photosensitizer undergoes electron- or hydrogen-transfer reactions with nearby substrates and/or molecular oxygen, generating radical species such as superoxide radical anion (O_2_˙^−^), hydroxyl radical (OH˙), and related oxidants. In Type II photosensitization, the triplet photosensitizer transfers its energy directly to ground-state triplet oxygen (^3^O_2_), producing singlet oxygen (^1^O_2_) and recovering the photosensitizer in its ground state. The balance between these pathways is highly relevant because it defines the nature, localization, and severity of oxidative DNA damage.^26^ These pathways differ in oxygen dependence, lifetime, diffusion range, and damage selectivity.^27^ Within a G4 complex, their relative contribution can be shaped by ligand aggregation, binding geometry, local polarity, proximity to stacked guanines, and guanine-mediated charge transfer.^12,28–31^ Thus, the folded nucleic acid scaffold may determine not only where a photosensitizer binds, but also how its excitation energy is converted into chemical damage.

In genomic G4s, such chemically directed damage may be therapeutically useful. Photooxidation of promoter or regulatory G4 sequences can introduce localized oxidative lesions, perturb G4 folding, interfere with transcription-factor recruitment, and alter oncogene-associated regulatory programs.^28,30–33^ In this context, G4 structural disruption is part of the mechanism by which a photoactive ligand converts selective recognition into localized genotoxic stress.^12,28,34^

A different problem emerges for G4 aptamers, which are synthetic, structure-defined oligonucleotides selected to recognize specific molecular targets through their folded three-dimensional architecture.^35^ In this case, the nucleic acid fold is not only a photochemical target, but also the molecular-recognition element that enables biological function. AS1411 is a prototypical example.^36^ This guanine-rich DNA aptamer adopts polymorphic G4 architectures and binds nucleolin (NCL), a multifunctional protein enriched in nucleolar and nuclear compartments and aberrantly displayed at the plasma membrane of many cancer cells.^36–38^ AS1411 reached phase II clinical evaluation in relapsed/refractory acute myeloid leukemia and metastatic renal cell carcinoma.^37,38^ Its nuclease resistance, clinical history, and compatibility with drugs, nanoparticles, and imaging probes have established AS1411 as one of the most influential G4-based scaffolds for cancer-associated delivery and molecular targeting.^38–42^

The targeting potential of AS1411 has also been extended to photodynamic platforms (Scheme 1A). AS1411 has been combined with porphyrin-type photosensitizers, including TMPyP4, to promote cancer-cell targeting, fluorescence activation, ROS generation, and photodynamic activity.^43–46^ However, these studies mainly treated AS1411 as a targeting or carrier element. They did not resolve how the folded aptamer architecture controls the photochemical mechanism of a bound photosensitizer, where oxidative chemistry occurs within the aptamer, or whether light-induced guanine oxidation preserves or compromises NCL recognition.

**Scheme 1:**
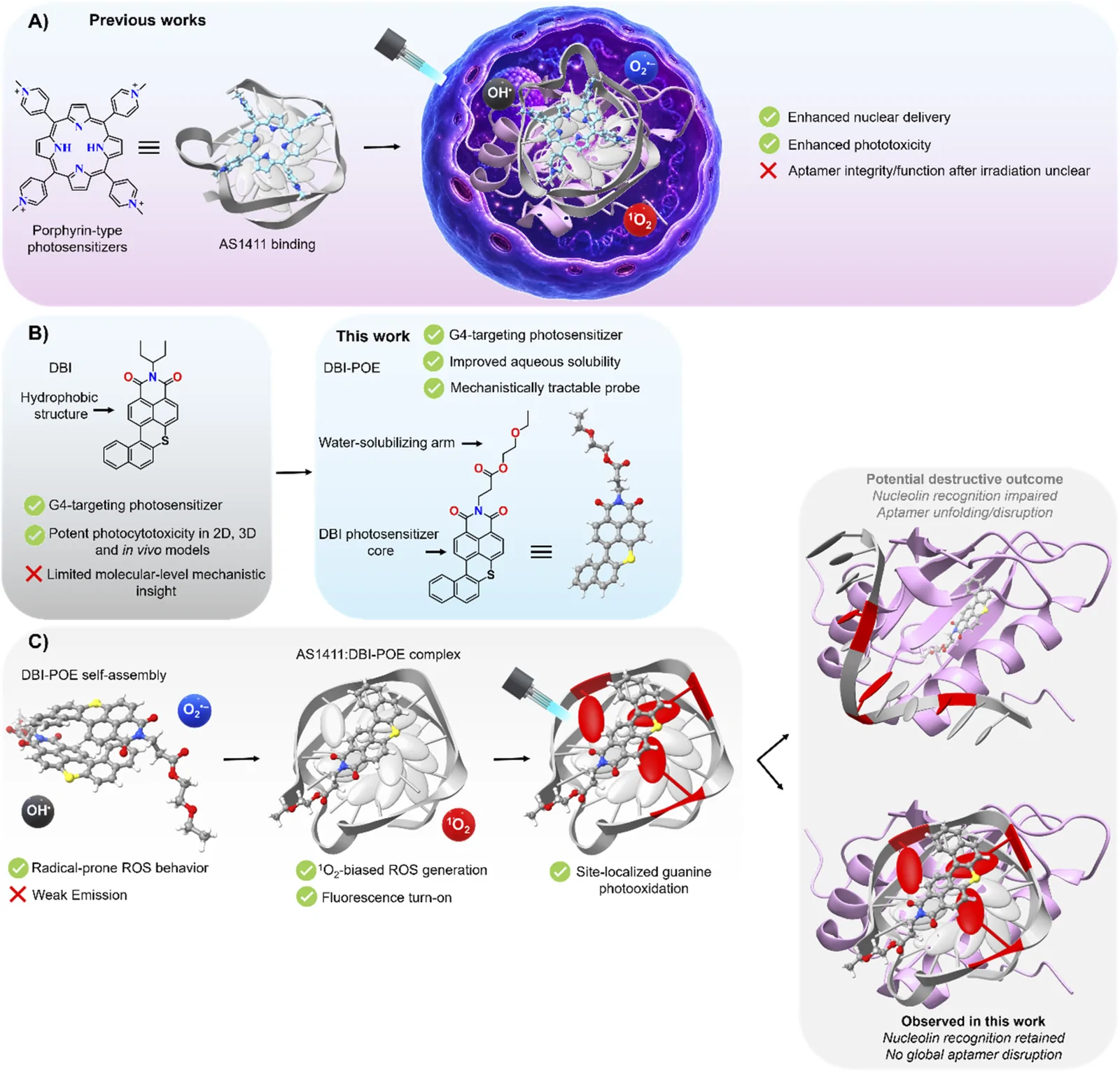
(A) Previous AS1411–photosensitizer systems have enhanced nuclear delivery and photocytotoxicity, but the fate of the aptamer after photoactivation has remained unclear. (B and C) Here, DBI-POE is developed as a water-solubilized and mechanistically tractable DBI-derived G4-targeting photosensitizer. AS1411 binding switches DBI-POE from a weakly emissive, radical-prone self-assembled state to a fluorescent G4-bound complex with ^1^O_2_-biased ROS generation, enabling site-localized guanine photooxidation without global aptamer disruption or loss of NCL recognition.

This unresolved point creates a specific design problem for aptamer–photosensitizer systems. Association with AS1411 could improve selectivity by exploiting NCL-mediated recognition of cancer-associated cells and by concentrating photoactive cargo in NCL-rich nuclear or perinuclear environments. After light activation, however, the fate of the aptamer becomes decisive. In one regime, ROS generation damages the guanine-rich AS1411 fold, promotes unfolding, and weakens or eliminates NCL recognition. In a second regime, the folded aptamer confines ROS generation or charge-transfer events to selected guanines while retaining the structural features required for protein binding. Both outcomes are chemically plausible, but they imply fundamentally different design principles. Distinguishing between them requires a molecular-level analysis that connects ROS identity, site-resolved guanine oxidation, structural persistence, and protein recognition within the same activated complex.

To interrogate this problem, we used DBI-POE, a DBI-derived heavy-atom-free dibenzothioxanthene imide amphiphilic photosensitizer that combines G4 recognition, reversible aqueous self-assembly, fluorescence responsiveness, and ROS generation (Scheme 1B).^12,32,33,47^ Previous work showed that DBI-POE undergoes G4-controlled photochemical switching: in water, it forms weakly emissive aggregates associated with radical-rich Type I reactivity, whereas binding to the MYC promoter G4 restores fluorescence, promotes ^1^O_2_ generation, and induces site-selective guanine oxidation accompanied by G4 unfolding.^12^ This precedent raised a decisive question: does AS1411 enforce the same destructive photochemical outcome, or can its aptameric G4 architecture redirect DBI-POE reactivity toward localized oxidation while preserving a recognition-competent structure?

Here, we show that AS1411 acts as an active photochemical scaffold for DBI-POE rather than as a passive carrier. The folded aptamer converts DBI-POE from a weakly emissive, radical-prone aggregate into a fluorescent G4-bound complex with a predominantly ^1^O_2_-associated photochemical regime and strongly suppressed radical-probe responses. ^1^H NMR spectroscopy, molecular dynamics (MD) simulations, and comparison of experimental and quantum-mechanically (QM) calculated circular dichroism (CD) spectra are consistent with a preferential DBI-POE binding mode at the 3′-terminal tetrad of an AS1411-derived G4 model, while primer-extension experiments independently identify light-induced guanine oxidation at this site with single-nucleotide resolution. Unfolded AS1411 in Li^+^ buffer and a G4-disrupted mutant fail to reproduce this damage pattern, demonstrating that folded architecture, not guanine content alone, dictates oxidative selectivity. Crucially, this localized photooxidation does not erase the BG4-recognized G4 state and does not abolish apparent binding to full-length NCL. These findings establish a function-preserving mode of G4 photosensitization in which light-triggered chemical damage and biological recognition remain compatible within the same folded nucleic-acid scaffold (Scheme 1C).

## Results and discussion

We first examined the intrinsic supramolecular behavior of DBI-POE in aqueous solution, because aggregation is expected to influence both fluorescence and ROS generation. At low temperature, DBI-POE displayed an aggregate-associated absorption profile with an intense band at 502 nm and a red-shifted shoulder near 540 nm (Fig. 1A).^12^ This spectral shape is consistent with excitonically coupled assemblies, most likely slipped π-stacked dimers or higher-order aggregates.^48^ In this state, fluorescence was strongly suppressed, indicating efficient nonradiative deactivation through aggregate-caused quenching (ACQ), intermolecular electronic coupling, and/or charge-transfer interactions between closely packed chromophores. Upon heating, the absorption spectrum evolved toward a more monomer-like profile, with a clear isosbestic point and a pronounced fluorescence enhancement centered at 564 nm (Fig. 1A).^49^ Absorption and fluorescence measurements were performed at 10 and 2 µM, respectively, to ensure adequate absorbance signal-to-noise while minimizing inner-filter and reabsorption effects in fluorescence measurements. These changes indicate a clean interconversion between a quenched aggregated state and a more emissive disassembled state.^50^ Thus, even in the absence of DNA, DBI-POE exhibits thermally regulated supramolecular behavior, in which fluorescence is controlled by the equilibrium between aggregated and disassembled species.^12^

**Fig. 1:**
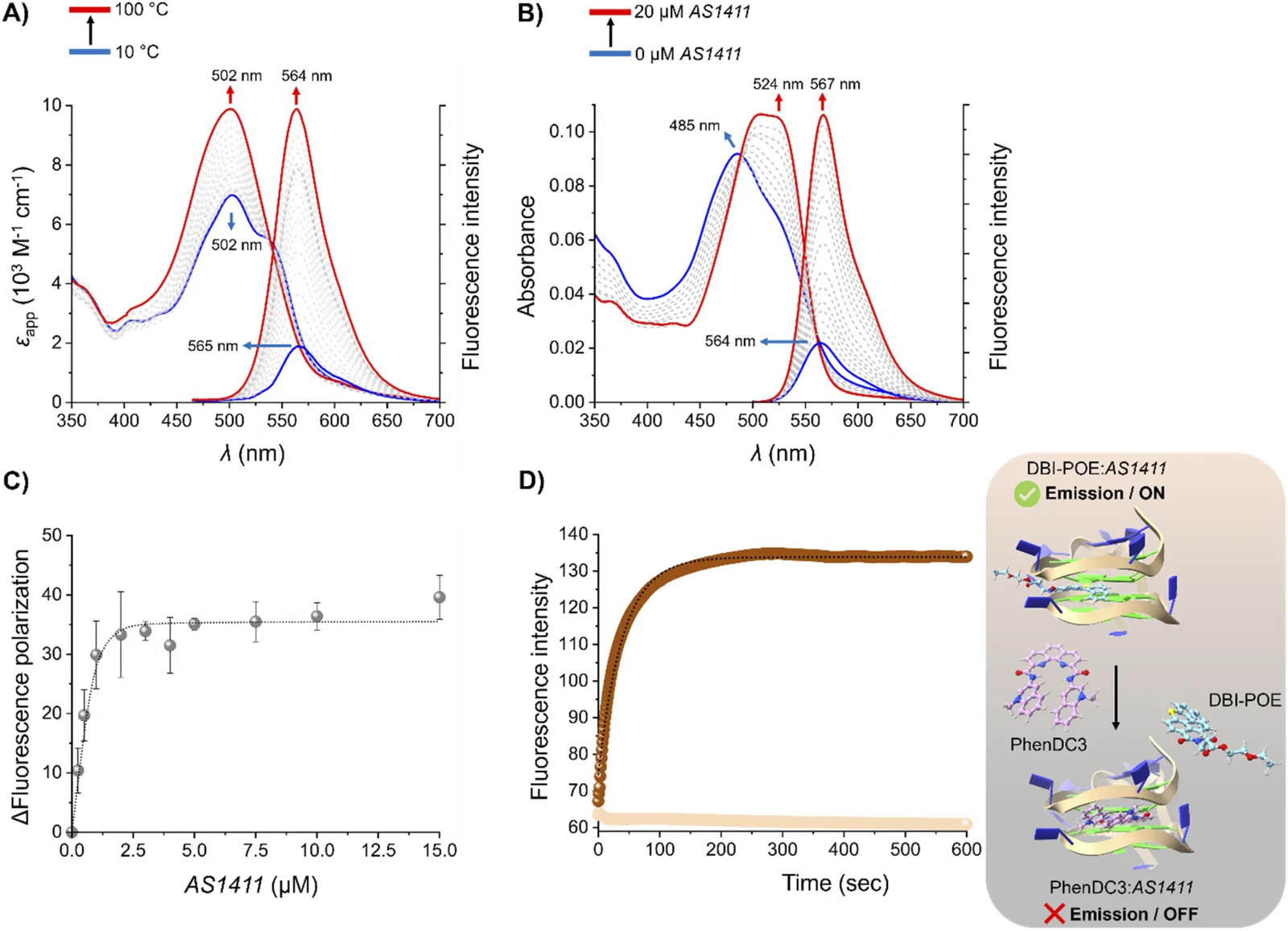
(A) Temperature-dependent UV-vis absorption and fluorescence emission spectra of DBI-POE in H_2_O recorded from 10 to 100 °C. Spectra at 10 °C and 100 °C are shown in blue and red, respectively, with intermediate temperatures in gray. DBI-POE was used at 10 µM for absorption and 2 µM for emission; *λ*_exc_ = 460 nm. (B) UV-vis absorption and fluorescence emission changes of DBI-POE upon AS1411 binding. DBI-POE alone is shown in blue, and spectra recorded with increasing AS1411 concentrations are shown from gray to red. Measurements were performed in 50 mM Tris buffer, pH 6.8, containing 100 mM KCl, using 10 µM DBI-POE for absorption and 2 µM for emission; AS1411 was titrated from 0 to 20 µM; *λ*_exc_ = 489 nm. (C) Fluorescence polarization analysis of DBI-POE binding to AS1411. DBI-POE was used at 1 µM and titrated with AS1411 from 0 to 15 µM; *λ*_exc_/*λ*_em_ = 482 ± 16/530 ± 40 nm. The dashed line shows nonlinear fitting using a 1 : 1 binding model. Data are the mean ± SD of three independent experiments. (D) Time-dependent competition assay with PhenDC3. DBI-POE, AS1411, and PhenDC3 were each used at 2.0 µM; *λ*_exc_ = 489 nm. The dashed line shows single-exponential fitting, giving an apparent time constant *τ*_app_ = 39.9 ± 0.3 s. Inset: schematic of PhenDC3 competing with DBI-POE for AS1411 binding.

We next asked whether this equilibrium could be controlled by a biologically relevant G4 aptamer.^51^ Native AS1411 was compared with three controls designed to separate structure-dependent recognition from nonspecific nucleic-acid effects: the complementary C-rich strand, a G4-disrupted AS1411 mutant, and a self-complementary duplex DNA (Table S1). CD confirmed that AS1411 adopts a parallel-like G4 signature, with a positive band near 260 nm and a negative band near 240 nm (Fig. S1A).^51^ By comparison, the C-rich and G-to-C mutant controls showed less intense CD signals and did not exhibit the characteristic spectral features associated with G4 folding, consistent with their non-G4 character under these conditions (Fig. S1A). This structural assignment was further supported by ^1^H NMR spectroscopy, which revealed guanine imino proton resonances only for AS1411, consistent with the formation of hydrogen-bonded G-tetrads (Fig. S1B).^51^ BG4 dot-blot recognition further supported formation of a folded G4 state by AS1411 (Fig. S1C). Primer-extension experiments provided a functional readout of G4 formation. In 50 mM K^+^, only AS1411 impeded polymerase progression, whereas AS1411 in 50 mM Li^+^ and the non-G4 controls allowed predominantly full-length extension, consistent with reduced formation of a polymerase-blocking G4 barrier (Fig. S1D). Together, these orthogonal assays establish that the subsequent DBI-POE response can be interpreted in relation to a folded G4 architecture rather than to guanine richness or polyanionic DNA binding alone.

Addition of AS1411 to DBI-POE in K^+^ buffer produced a pronounced reorganization of the absorption spectrum (Fig. 1B). The aggregate-associated absorption profile developed into a hyperchromic, well-resolved band centered at 524 nm, consistent with formation of a defined AS1411-bound species.^12,52–54^ The presence of an isosbestic point supports a dominant transition between aggregated DBI-POE and a G4-associated state rather than a broad distribution of nonspecific complexes. Molecular docking and classical MD simulations (Fig. S15–S18 and Table S2), followed by QM calculations on representative binding poses (Fig. S22), supported this assignment by showing that the computed absorption profiles of the G4-bound models (Fig. S23 and Table S3) reproduce the main experimental spectral features. This spectral conversion was accompanied by an approximately five-fold fluorescence enhancement, showing that AS1411 releases DBI-POE from a self-quenched aggregated state and stabilizes an emissive bound form.^12,52–54^ In agreement with this interpretation, QM calculations on the AS1411-bound model showed that the optimized S_1_ minimum retains the key features of the previously described emissive G4-bound state, supporting a conserved fluorescence-activation mechanism within the AS1411 scaffold (Fig. 2A and Table S4).^12^ By contrast, the non-G4 controls produced only minor fluorescence recovery and weaker absorption changes, indicating that nonspecific nucleic-acid association can perturb DBI-POE aggregates but does not generate the same productive emissive complex (Fig. S2–S3).^12^

**Fig. 2:**
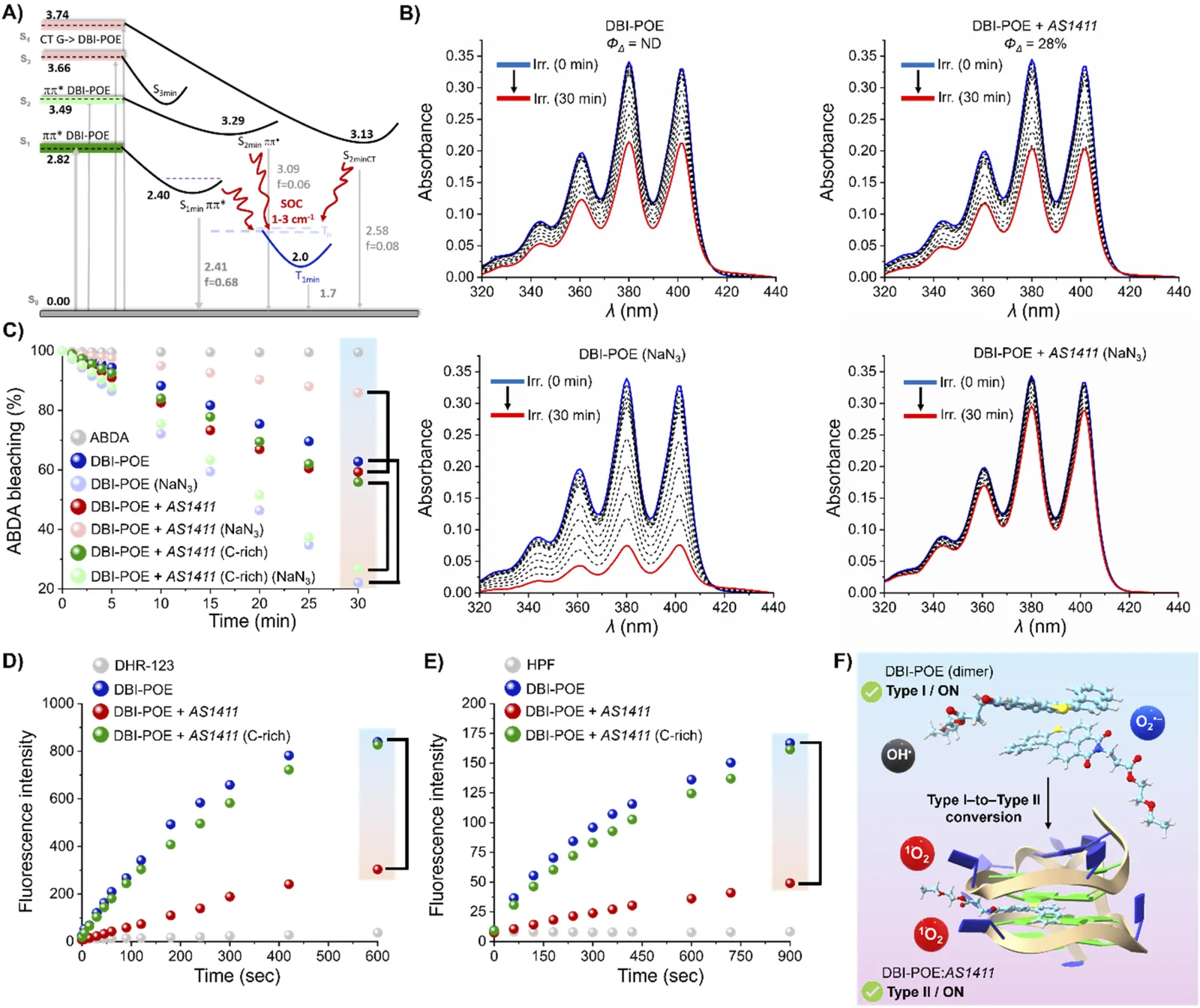
(A) Computed singlet and triplet potential-energy surfaces of DBI-POE in the 3′-end-associated intercalated binding mode of the G4 aptamer. Adiabatic energies are reported in eV relative to the ground state at the Franck–Condon geometry; oscillator strengths and vertical energies are indicated in gray for the relevant singlet transitions. (B) ABDA bleaching assay. Samples contained ABDA (30 µM), DBI-POE (5 µM), and, where indicated, AS1411 or AS1411 C-rich control (5 µM), in 50 mM Tris buffer, pH 6.8, containing 100 mM KCl. NaN_3_ (10 mM) was added where indicated. (C) ABDA bleaching monitored at 380 nm and expressed as percentage. (D and E) Fluorescence enhancement of DHR-123 (D; *λ*_exc_ = 488 nm) and HPF (E; *λ*_exc_ = 490 nm). Samples contained the corresponding probe (5 µM), DBI-POE (5 µM), and, where indicated, AS1411 or AS1411 C-rich control (5 µM), in the same buffer as in B. (F) Schematic representation of the photochemical behavior of DBI-POE in the free and AS1411-bound states, highlighting the G4-dependent change in ROS output. In all experiments, samples were irradiated at 485 ± 20 nm for the indicated time intervals.

Binding analysis reinforced this interpretation. Job plot experiments were consistent with a predominant 1 : 1 DBI-POE : AS1411 complex, while fluorescence polarization gave an apparent association constant (*K*_a,app_) of 8.3 × 10^7^ M^−1^ under the experimental conditions used (Fig. 1C and S4). To further probe whether DBI-POE fluorescence activation originates from binding to a defined G4 interface, we performed competition experiments with PhenDC3, a benchmark high-affinity G4 ligand.^55,56^ PhenDC3 caused a pronounced decrease in the G4-associated DBI-POE fluorescence response, consistent with competition for the G4 binding environment (Fig. 1D).

We then investigated whether AS1411 binding modulates the photochemical output of DBI-POE. To connect the experimentally observed G4-induced fluorescence turn-on with ROS generation, we analyzed the computed singlet and triplet potential-energy surfaces of the 3′-end-associated intercalated DBI-POE model (Fig. 2A). Docking/MD sampling identified several energetically accessible binding arrangements, and comparison of the QM-calculated CD spectra of representative structures with experiment provided the strongest qualitative support for the 3′-intercalated model among the geometries considered (Fig. S23B and Table S2). Independent experimental evidence described below, including NMR perturbation mapping and site-resolved oxidative-damage analysis, further supports preferential DBI-POE engagement and photochemical reactivity at the same 3′-terminal region. Together, these complementary observations are consistent with a 3′-end-associated, partially intercalated binding model, without establishing a unique atomic-resolution pose. The calculated excited-state landscape shows that the optimized singlet minimum is potentially emissive (*f* ∼ 0.7) and connected to nearby triplet (T) states through non-negligible spin–orbit coupling (SOC), supporting an accessible intersystem-crossing (ISC) pathway (Table S5). Subsequent relaxation on the triplet surface yields a stable T_1_ minimum at approximately 1.7 eV, well above the ∼0.98 eV energy required to generate ^1^O_2_, indicating that the AS1411-bound DBI-POE complex is energetically competent for Type II photosensitization.^57^ Thus, the calculations indicate that the AS1411-bound DBI-POE complex is not only fluorescence-activated within the G4 scaffold, but also retains a triplet excited state energetically competent for Type II photosensitization.

We therefore tested whether this predicted photochemical competence translated into ROS production experimentally. Upon irradiation, DBI-POE induced progressive bleaching of 9,10-anthracenediyl-bis(methylene)dimalonic acid (ABDA), a water-compatible anthracene probe commonly used to monitor ^1^O_2_-associated oxidation, indicating the formation of photoinduced oxidizing species capable of consuming the probe (Fig. 2B, C and S5). DBI-POE produced comparable ABDA bleaching in the presence of AS1411 and the C-rich control, showing that photooxidative activity is retained under both oligonucleotide-binding conditions.^12^

To gain mechanistic insight into the nature of this process, we examined the effect of sodium azide (NaN_3_), one of the most widely used physical quenchers of ^1^O_2_ in photochemical and photodynamic studies.^12,58–60^ In the DBI-POE complex, NaN_3_ strongly suppressed ABDA bleaching (∼66%), consistent with a substantial NaN_3_-sensitive, ^1^O_2_-associated contribution to probe oxidation (Fig. 2C and S5). By contrast, NaN_3_ did not suppress ABDA bleaching for DBI-POE alone or for DBI-POE in the presence of the C-rich control; instead, it enhanced the bleaching response.^12^ Although NaN_3_ is widely employed as a ^1^O_2_ quencher, previous studies have shown that its behavior can become more complex in radical-rich photosensitizing systems. Huang *et al.* reported a paradoxical enhancement of methylene-blue-mediated photodynamic activity in the presence of NaN_3_ and directly detected formation of the azidyl radical (
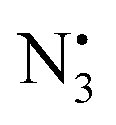
) by spin-trapping/EPR experiments.^60^ In a related study, NaN_3_ enhanced hydroxyphenyl fluorescein (HPF) activation during Type-I photosensitization and Fenton chemistry, which was attributed to secondary azidyl-radical chemistry.^61^ Notably, differential NaN_3_ responses have also been reported in ABDA-based photosensitization assays, where NaN_3_ efficiently suppressed ABDA consumption for predominantly Type-II photosensitizers, whereas substantial probe bleaching remained in systems displaying an additional Type-I contribution.^62^ Consistently, NaN_3_ has also been employed in mixed Type-I/Type-II photosensitizing systems to suppress the ^1^O_2_ contribution while radical generation remained essentially unaffected.^63^

This divergent behavior indicates that, outside the folded AS1411 G4 environment, DBI-POE operates through a different photochemical regime that is not dominated by simple ^1^O_2_-mediated ABDA oxidation. Rather, the NaN_3_-dependent enhancement is consistent with a radical-rich Type I pathway, in which azide may intercept photoinduced oxidizing equivalents or radical intermediates and generate secondary azidyl-radical processes that further contribute to ABDA oxidation.^60,64^ Accordingly, the NaN_3_-dependent enhancement is interpreted here as being consistent with, rather than direct evidence for, secondary azide-mediated radical chemistry. Importantly, ABDA remained unchanged when irradiated with NaN_3_ in the absence of DBI-POE, excluding direct ABDA/azide photoreactivity as the origin of the observed effects.

This divergent NaN_3_ response provides a useful mechanistic signature. In the absence of a folded G4 scaffold, DBI-POE remains in a more aggregate-associated and radical-prone photochemical regime, likely promoted by intermolecular electron transfer and inefficiently controlled excited-state deactivation. In contrast, AS1411 binding converts DBI-POE into a G4-associated photosensitizer whose ABDA response becomes NaN_3_-sensitive, indicating a shift toward predominantly Type II-like ^1^O_2_ production.^12^ This assignment was further supported by a ^1^O_2_ quantum yield (*Φ*_Δ_) of 28% for the DBI-POE : AS1411 complex, a substantial value for a heavy-atom-free photosensitizer operating in aqueous buffer. Because ABDA bleaching provides an indirect probe-based readout of ^1^O_2_-associated oxidation, and single-probe assays may be insufficient to fully distinguish Type II from Type I photochemical pathways in complex photosensitized systems, we complemented this assay with radical-sensitive probes. HPF, which reports highly reactive oxidants such as OH˙, and dihydrorhodamine 123 (DHR-123), a redox probe commonly used to monitor O_2_˙^−^-associated oxidation, both gave markedly weaker responses for the DBI-POE complex than for DBI-POE alone or DBI-POE with the C-rich control (Fig. 2D–E).^26,65^ Relative to DBI-POE alone, AS1411 complexation reduced the DHR-123 and HPF responses by approximately 64% and 71%, respectively. Importantly, this ROS redistribution provides a direct mechanistic link to the site-localized damage pattern discussed below. Guanine is the most readily oxidized canonical nucleobase and a preferred target of ^1^O_2_-mediated DNA oxidation, whereas highly reactive radical pathways, particularly those involving OH˙, are generally less selective and can promote broader base and sugar damage.^66–68^ Thus, AS1411 does not merely activate DBI-POE; it reshapes its excited-state output toward a ^1^O_2_-biased pathway that is chemically suited to localized guanine oxidation within the folded G4 scaffold rather than diffuse aptamer damage (Fig. 2F).

Having established that AS1411 modulates DBI-POE photochemistry, we asked whether this light-triggered reactivity damages the aptamer scaffold itself. As a control, irradiation of AS1411 in the absence of DBI-POE did not appreciably alter its CD profile, indicating that the aptamer is not measurably perturbed by light exposure alone under these conditions (Fig. S6). In the dark, DBI-POE caused only minor changes in the AS1411 CD spectrum, suggesting that ligand binding does not substantially remodel the folded aptamer (Fig. S7). Consistently, simulated CD spectra obtained from docking/MD-derived DBI-POE models showed that the chiroptical response depends strongly on the binding geometry (Fig. S23B). Notably, based on the available CD data, the 3′-intercalated arrangement appears to provide the best qualitative agreement with the experimental spectral features, including the negative–positive–negative pattern observed in the measured region. These data indicate that DBI-POE binding is compatible with preservation of the global G4 fold and favors a partially inserted pose within the 3′-terminal G-quartet region. By contrast, irradiation of the DBI-POE : AS1411 complex led to a gradual decrease in CD amplitude, consistent with chemical modification, increased conformational heterogeneity, and/or partial perturbation of the G4 ensemble (Fig. 3A).^12,13,69,70^ However, this loss of CD intensity did not correspond to complete unfolding. CD melting experiments showed that DBI-POE moderately stabilized AS1411 in the dark, with a Δ*T*_m_ of approximately 3 °C, while irradiated samples retained melting transitions close to those of native AS1411 even after prolonged exposure (Fig. 3B and S8). Thus, irradiation weakens the optical signature of the folded ensemble but does not abolish thermal behavior characteristic of a G4 state.

**Fig. 3:**
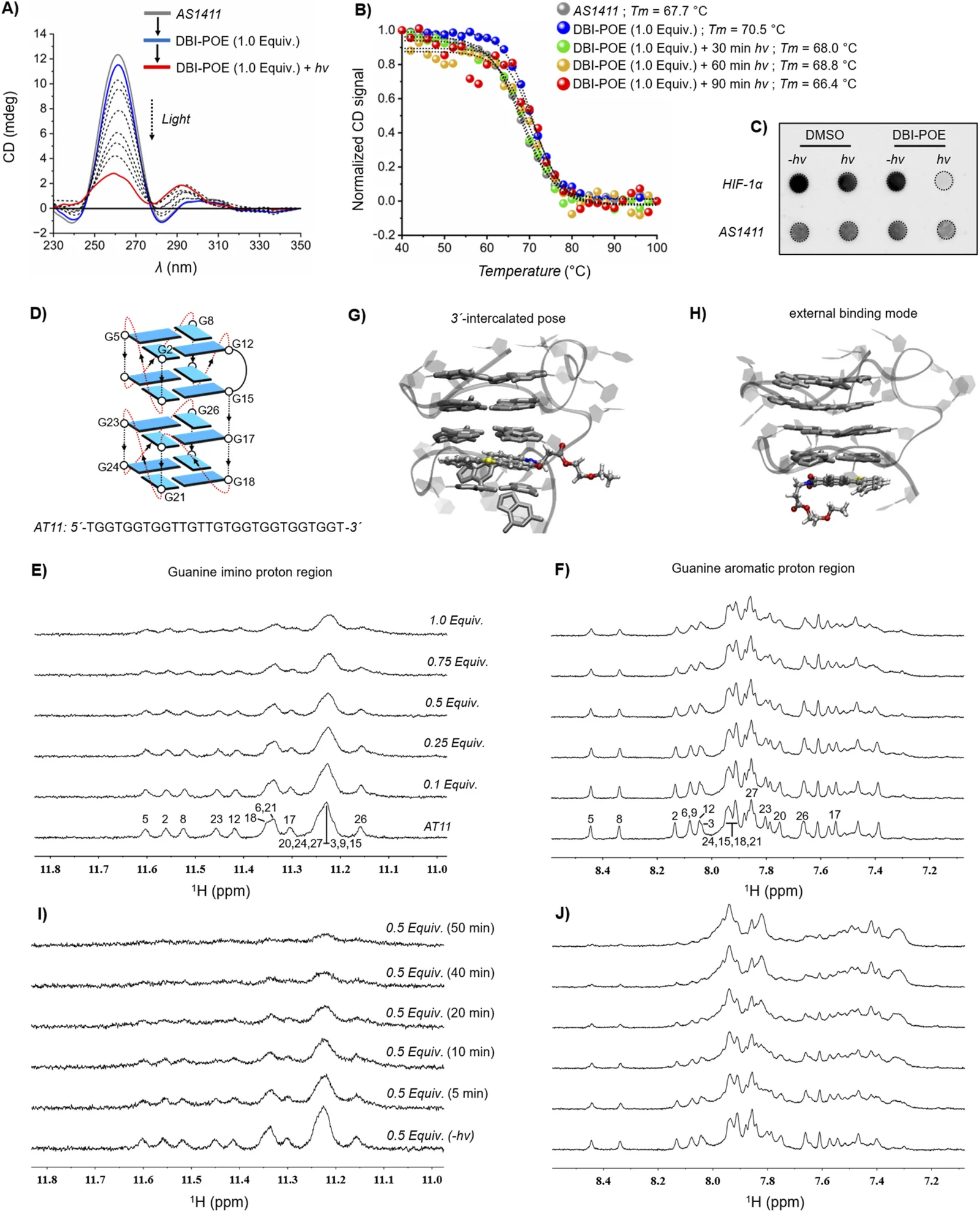
(A) CD spectra of AS1411 recorded in the absence or presence of DBI-POE after irradiation for up to 60 min. Measurements were performed using AS1411 (2 µM) and DBI-POE (2 µM) in 10 mM Tris buffer, pH 6.8, containing 100 mM KCl. (B) Normalized CD-monitored melting profiles of AS1411, recorded at 262 nm, in the absence or presence of DBI-POE under different irradiation conditions. Measurements were performed using AS1411 (2 µM) and DBI-POE (0 or 2 µM) in the same buffer as in A. (C) BG4-based dot blot analysis of HIF-1α and AS1411 G4-forming sequences in the absence or presence of DBI-POE under dark and irradiated conditions, performed in 10 mM Tris buffer, pH 7.4, containing 100 mM KCl. (D) Schematic representation of the G-tetrad alignment and folding topology of the AT11 G4. (E and F) ^1^H NMR spectra of the guanine imino (E) and aromatic (F) regions of AT11 upon titration with DBI-POE. Measurements were performed using AT11 (350 µM) and DBI-POE from 0 to 350 µM in 10 mM Tris buffer, pH 6.8, containing 70 mM KCl. (G and H) Representative MD clusters for the 3′-intercalated (G) and external (H) DBI-POE binding modes. (I and J) ^1^H NMR spectra of the guanine imino (I) and aromatic (J) regions of the AT11 complex after different irradiation intervals. Measurements were performed using AT11 (350 µM) and DBI-POE (175 µM) in the same buffer as in E and F.

Because CD amplitude alone cannot distinguish global unfolding from local base oxidation, BG4 dot-blot experiments were used as an independent structural readout.^12^ BG4 is a structure-selective antibody fragment that binds a broad range of G4 topologies; however, recent studies indicate that its signal can persist even when G4s contain destabilizing single-base substitutions or oxidative lesions such as 8-oxoG, although binding strength may depend on lesion position and the extent of structural perturbation.^71,72^ Therefore, a retained BG4 signal should not be taken as evidence that AS1411 remains chemically intact, but rather that an antibody-detectable G4 epitope is still present after oxidation. This point was underscored by the contrasting behavior of the two G4 scaffolds: DBI-POE-mediated irradiation strongly reduced the BG4 signal of the HIF-1α promoter G4 positive control, whereas AS1411 maintained robust antibody binding after irradiation (Fig. 3C).^12^ Thus, the structural outcome of photooxidation is scaffold-dependent. In HIF-1α, oxidative damage appears to disrupt the G4 architecture required for BG4 binding, whereas in AS1411, chemical modification occurs without eliminating the folded epitope. This divergence is central to the study, showing that G4 architecture controls not only photosensitizer binding and ROS identity, but also whether oxidative chemistry compromises or preserves the folded state required for aptamer-guided function.

To locate the preferred DBI-POE binding site, we performed ^1^H NMR titrations using AT11, a structurally defined AS1411-derived sequence (Fig. 3D).^42,51^ Full-length AS1411 is known to populate multiple G4 conformations in solution, which complicates residue-level structural analysis.^51^ AT11 was developed to overcome this limitation by favoring a single major G4 conformation while retaining biological activity comparable to AS1411.^51^ Productive DBI-POE binding to AT11 was first confirmed by the characteristic fluorescence enhancement, while CD spectroscopy showed preservation of the overall G4 spectral signature upon DBI-POE binding in the dark. Light irradiation induced more pronounced CD changes, comparable to those observed for AS1411, consistent with photoinduced perturbation of the folded ensemble without complete loss of the G4 scaffold (Fig. S9). Its NMR solution structure revealed a four-layer, right-handed parallel G4 composed of two stacked propeller-type subunits connected by a thymine linker.^51^ This architecture provides an unusually well-defined framework for interpreting ligand-induced perturbations because the guanine imino and aromatic protons were assigned and the individual G-tetrads are structurally resolved.^51^ Upon DBI-POE addition, the strongest imino-proton perturbations clustered around guanines forming the 3′-terminal tetrad region, especially G18, G21, and G24 (Fig. 3E). These residues belong to the terminal tetrad of the second propeller-type block, together with G27, placing the largest spectral response at a solvent-exposed G-quartet face rather than at the internal stacking interface between the two subunits. Because resonance overlap and line broadening prevent fully quantitative site assignment, these data are interpreted as being consistent with preferential DBI-POE engagement of the 3′-terminal region, rather than as defining a unique binding geometry. The aromatic proton region supported the same conclusion. The persistence of the overall aromatic envelope indicates that DBI-POE binding does not globally affect the AT11 fold (Fig. 3F). At the same time, selected resonances, particularly those associated with the 3′-terminal region, were selectively reshaped or broadened. Importantly, the NMR data alone do not distinguish between a purely external end-stacking pose and a more inserted 3′-end-associated geometry. Docking/MD sampling identified both arrangements as energetically accessible, with the representative 3′-intercalated and external binding modes displaying interaction energies of −63.90 and −37.94 kcal mol^−1^, respectively (Fig. 3G–H and Table S2). Comparison of the QM-calculated CD spectra of representative structures with experiment further provided the strongest qualitative support for the 3′-intercalated model among the geometries considered (Fig. S23B). Irradiation of the DBI-POE : AT11 complex caused progressive broadening of imino signals, consistent with local oxidation, increased conformational exchange, or destabilization of hydrogen-bonded guanine environments (Fig. 3I).^12,69,70^ Importantly, although 3′-terminal tetrad resonances showed pronounced broadening, aromatic signals remained detectable after extended irradiation, arguing against complete destruction of the aptamer scaffold (Fig. 3J). The aromatic-region changes were most evident for resonances associated with the second stacked propeller-type subunit, further supporting local DBI-POE engagement and photooxidation near the 3′-terminal region while preserving much of the global G4 framework.

To connect this structural model to the precise sites of chemical damage, we next used primer-extension assays, which provide a functional readout of both G4-dependent polymerase arrest and lesion-induced pausing.^12,73^ In this assay, a DNA polymerase extends a primer along AS1411 or AT11 templates until progression is impeded by a folded G4 barrier or by an oxidized base that reduces bypass efficiency.^12,74^ Because polymerase arrest is typically detected one nucleotide before the obstructing G-tract or lesion site, the position of each pause reports the nucleotide immediately preceding the structural or chemical block.^6^ The assay was performed under optimized KCl conditions: different KCl concentrations were screened, and 10 mM KCl was selected as the best compromise to maintain a stable G4 fold while avoiding complete polymerase blockage, thereby allowing lesion-dependent changes in the primer-extension assay to be resolved (Fig. S10). Under dark conditions, the primer-extension profile obtained in the presence of DBI-POE was broadly similar to the control sample lacking DBI-POE, with only a modest reduction in full-length product and a dominant pause one nucleotide before the expected G4-forming tract (Fig. 4A–C). This indicates that ligand binding alone does not substantially alter polymerase processivity under these conditions. Upon irradiation, however, the full-length product was strongly suppressed and three new pause bands appeared, and their intensities increased with irradiation time. These stops occurred at T25, T22, and T19, corresponding to polymerase arrest immediately before G24, G21, and G18, respectively (Fig. 4A and B). At the same time, the intensity of the dominant G4-associated pause at T28 decreased, suggesting that photooxidation partially remodels or opens the original G4 barrier while generating new lesion-associated blocks within the 3′-terminal G-tract (Fig. 4C). The primer-extension pattern therefore identifies guanines within the 3′-terminal tetrad region as the major site of DBI-POE-mediated photooxidation and indicates that local chemical damage changes how the folded G4 is encountered by the polymerase. Importantly, a comparable irradiation-dependent pausing pattern, involving the corresponding guanine positions, was also observed with AS1411, providing an experimental bridge between the structurally tractable AT11 model and the parent aptamer and supporting transfer of the key site-localization conclusion to AS1411 (Fig. S11).

**Fig. 4:**
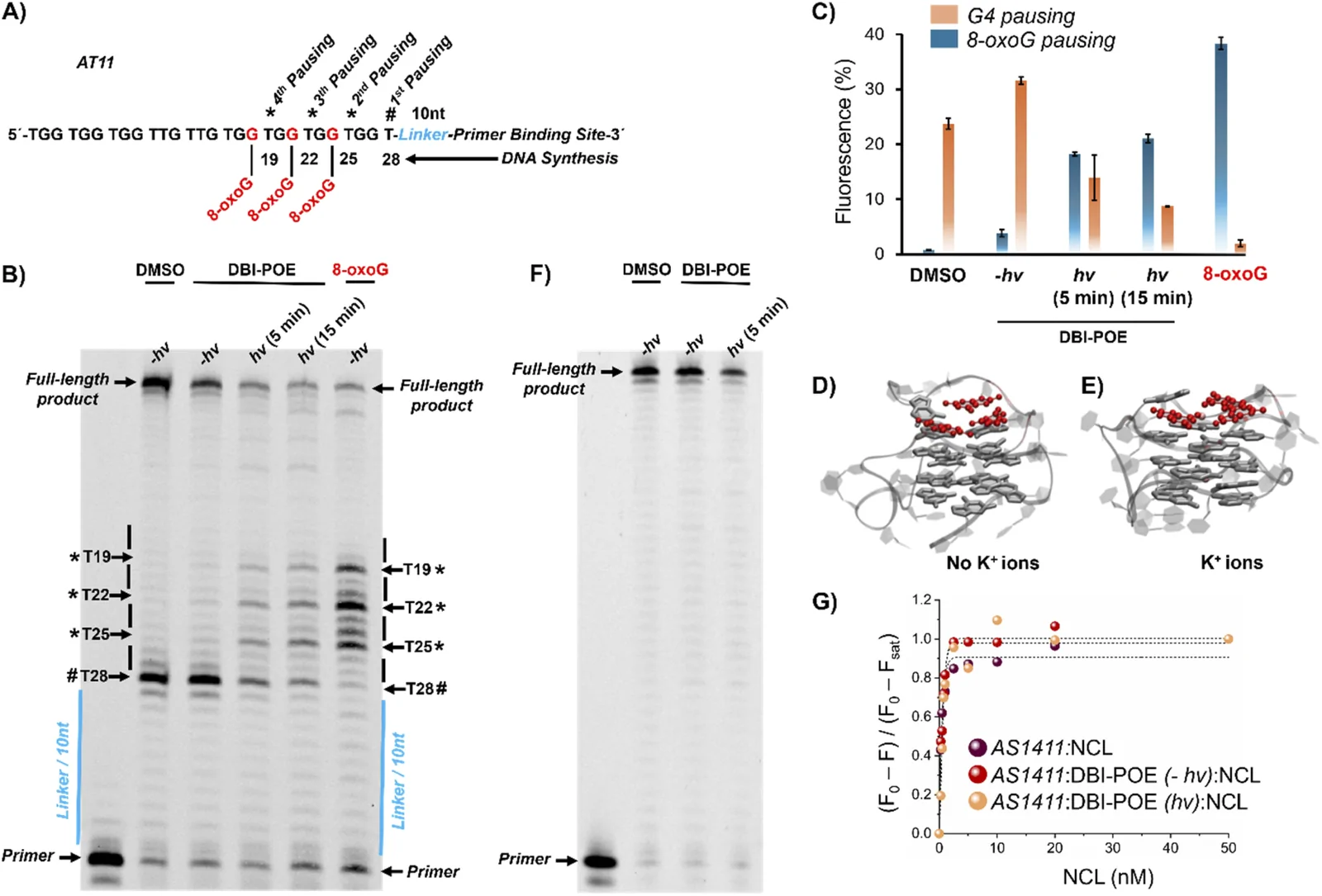
(A) AT11 template used for primer-extension analysis. Symbols indicate the polymerase pausing sites detected in B, and site-specifically incorporated 8-oxoG lesions are highlighted in red. (B) Representative primer-extension assays performed with AT11 under G4-stabilizing conditions in 10 mM KCl, using DBI-POE (1 µM). Black arrows indicate the non-extended primer, polymerase pausing sites, and full-length products. (C) Quantification of primer-extension pausing from B. G4-associated pausing was quantified at T28, whereas oxidative damage–mediated pausing was quantified as the summed intensity of the T25, T22, and T19 pause bands. The experiment was performed in duplicate; error bars represent absolute error. (D and E) Representative structures from the 1000–2000 ns MD simulation window of AT11 containing 8-oxoG (red) at positions 18, 21, and 24: (D) in the absence of intercalated K^+^ ions and (E) in the presence of intercalated K^+^ ions. (F) Representative primer-extension assays performed with AT11 under non-G4-stabilizing conditions in 50 mM LiCl. (G) Cy5 fluorescence-based NCL-binding profiles of AS1411, AS1411 : DBI-POE kept in the dark, and AS1411 : DBI-POE after irradiation. Experiments were performed using Cy5-labeled AS1411 (20 nM) and DBI-POE (20 nM), with NCL titrated from 0 to 50 nM in 50 mM Tris buffer, pH 6.8, containing 100 mM KCl; *λ*_exc_ = 630 nm. Data represent the average of two independent experiments.

To assign the chemical origin of these pauses, an AT11 template containing site-specific 8-oxoG substitutions at G24, G21, and G18 was prepared. The engineered 8-oxoG template reproduced the same primer-extension pattern observed after DBI-POE irradiation, supporting assignment of the photogenerated lesions to guanine oxidation at these positions (Fig. 4A–C).^12^ To assess the intrinsic effect of site-specific oxidation on the stability of the G4 scaffold, MD simulations were performed using AT11 models containing 8-oxoG at G18, G21, and G24 in the absence of DBI-POE (Fig. 4D–E and S19–S21). The oxidized G4 remained structurally stable during the simulations, particularly in the presence of intercalated K^+^ ions, which preserved the tetrad stacking and the overall quadruplex architecture. In the absence of intercalated K^+^ ions, the global G4 fold was still largely maintained over the simulated time window, although the 3′-terminal tetrad began to show signs of local disruption toward the end of the trajectory. These simulations indicate that the experimentally assigned 8-oxoG lesions do not necessarily trigger global G4 unfolding, but that K^+^ coordination is important for maintaining the integrity of the oxidized terminal tetrad. We note that the post-photooxidation binding state of DBI-POE was not experimentally determined, and persistent ligand association could influence the structural response of the oxidized G4. Accordingly, the simulations describe the intrinsic effects of the identified 8-oxoG lesions in the ligand-free G4 rather than the complete post-photochemical DBI-POE complex.

Having established that the assigned oxidative lesions are compatible with a K^+^-stabilized G4 architecture, the structural requirement for this damage pattern was tested under Li^+^ conditions, where AT11 or AS1411 does not form the same stable G4 architecture. In Li^+^, DBI-POE irradiation produced no lesion-associated pausing, and polymerase processivity remained largely intact (Fig. 4F and S12). Therefore, the observed oxidation pattern is not dictated simply by guanine sequence or by the intrinsic oxidation potential of individual bases. Instead, site-selective oxidation requires the folded G4 architecture, which positions DBI-POE at the 3′-terminal region and confines reactive excited-state chemistry to nearby guanines. The agreement between NMR perturbations, primer-extension pauses, site-specific 8-oxoG controls, and Li^+^ unfolding experiments defines a coherent structure–reactivity relationship: AS1411/AT11 architecture determines both where DBI-POE binds and where light-triggered guanine oxidation occurs.

Finally, we examined whether localized oxidation under the defined photochemical conditions used in this study compromises the biological recognition function of AS1411. Cy5-labeled AS1411 was titrated with full-length NCL under three conditions: native AS1411, AS1411 bound to DBI-POE in the dark, and AS1411 photooxidized in the presence of DBI-POE. A proximity-induced Cy5 quenching titration confirmed that DBI-POE can bind AS1411 at the nanomolar concentrations used in the NCL assay (Fig. S13). Importantly, under the tested 1 : 1 DBI-POE : AS1411 loading condition and irradiation protocol, all three systems retained comparable apparent binding to full-length NCL. Native AS1411 showed an apparent *K*_a_ = 2.26 × 10^8^ M^−1^ (*K*_d_ = 4.4 nM), while AS1411/DBI-POE kept in the dark showed *K*_a_ = 2.39 × 10^8^ M^−1^ (*K*_d_ = 4.2 nM). After photoirradiation, the AS1411/DBI-POE system retained a similar apparent affinity, with *K*_a_ = 2.74 × 10^8^ M^−1^ (*K*_d_ = 3.6 nM). These values fall within the nanomolar range and are comparable to reported AS1411 : NCL affinities, although differences in protein construct, labeling strategy, and assay format can shift the apparent K_d_ (Fig. 4G).^75,76^ Thus, DBI-POE binding and localized light-induced guanine oxidation do not abolish the ability of AS1411 to recognize NCL under the defined photooxidation regime examined here. We note that this experiment was designed to establish functional preservation under this specific loading ratio and irradiation condition and does not define the upper limits of photosensitizer loading or irradiation dose compatible with NCL recognition.

## Conclusions

Taken together, these results show that AS1411 is not a passive carrier for DBI-POE, but an active photochemical scaffold that reshapes the behavior of the photosensitizer. AS1411 binding converts DBI-POE from a weakly emissive, aggregate-prone system into a G4-associated photosensitizer with enhanced fluorescence and a distinct ROS profile dominated by ^1^O_2_-associated chemistry. Photoactivation then produces localized guanine oxidation at defined sites within the AT11/AS1411 G4 framework, while CD, BG4 dot-blot analysis, NMR spectroscopy, MD simulations, and NCL-binding assays indicate that the global folded architecture and biological recognition function are largely preserved under the defined photooxidation conditions examined here. Thus, AS1411 can organize photochemical reactivity in a spatially localized manner without causing global destruction of aptamer function.

More broadly, this work provides a mechanistic basis for developing aptamer-photosensitizer systems in which G4 DNA architecture controls not only target recognition, but also photochemical output and the structural consequences of irradiation. In the present system, AS1411 favors a function-preserving regime, where localized oxidative modification occurs while the folded, NCL-binding-competent scaffold remains largely intact. Future systems could exploit this principle in different ways: preserving the aptamer fold to maintain target-associated photosensitization, or deliberately designing more labile aptamer–photosensitizer assemblies in which photoinduced structural disruption promotes photosensitizer release, redistribution, or amplified damage within G4-rich cellular environments. Defining how these outcomes depend on aptamer architecture, ligand structure, binding site, and ROS identity will be important for transforming aptamer-guided photochemistry into programmable therapeutic strategies. This framework now provides the basis for ongoing cellular studies aimed at determining how aptamer integrity, NCL-mediated localization, photosensitizer retention or release, and light-triggered cytotoxicity are coupled in biological environments.

## Author contributions

M. V. Cottini, K. Saczuk, J. Trojnar, L. López-Pacios, D. Puchán Sánchez, A. H. G. David, and M. Dudek: data curation, investigation, validation, and formal analysis. I. Obi and N. Sabouri: formal analysis. J. J. Nogueira: formal analysis, methodology, resources and supervision. C. Cabanetos: formal analysis and supervision. L. Martínez-Fernández and J. Jamroskovic: investigation, methodology, validation, formal analysis, supervision, data curation, resources. M. Deiana: conceptualization, investigation, methodology, validation, visualization, formal analysis, supervision, data curation, project administration, resources, funding acquisition, and writing – original draft.

## Conflicts of interest

There are no conflicts to declare.

## Supplementary Material

SC-OLF-D6SC06144C-s001

## Data Availability

The data supporting the findings reported in this study are available within the article and its supplementary information (SI). Supplementary information: experimental procedures, spectroscopic and reactive oxygen species generation studies, DNA-binding and structural analyses, oligonucleotide sequences and sample preparation protocols, primer-extension and dot-blot assays, computational methods and results, and additional tables and figures. See DOI: https://doi.org/10.1039/d6sc06144c.
