# Supplementary material for: Function-preserving photooxidation of AS1411 aptamer by a G-quadruplex-guided photosensitizer
Source: Chem Sci. 2026 Oct 5. Online ahead of print. doi: 10.1039/d6sc06144c (PMC13659297; doi:10.1039/d6sc06144c)
Supplement: Supplementary file 1 [file SC-OLF-D6SC06144C-s001.pdf]

Supplementary Information (SI) for Chemical Science.
This journal is © The Royal Society of Chemistry

Supplementary Information

Function-Preserving Photooxidation of *AS1411* Aptamer by a G-Quadruplex-Guided Photosensitizer

*Maria V. Cottini,[a] Karolina Saczuk,[b] Jakub Trojnar,[b] Lucía López-Pacios,[c] Dario Puchán Sánchez,[d] Arthur H. G. David,[d] Ikenna Obi,[e] Nasim Sabouri,[e,f] Juan J. Nogueira,[c,g] Marta Dudek,[b] Clément Cabanetos,[d] Lara Martínez-Fernández,[*h] Jan Jamroskovic,[*a] and Marco Deiana[*b]*

[a] Department of Microbial Genetics, Slovak Academy of Sciences, Institute of Molecular Biology, Dubravska cesta 21, 845 51 Bratislava, Slovakia. E-mail: jan.jamroskovic@savba.sk

[b] Institute of Advanced Materials, Faculty of Chemistry, Wrocław University of Science and Technology, Wyb. Wyspiańskiego 27, Wrocław 50-370, Poland. E-mail: m.deiana@pwr.edu.pl

[c] Department of Chemistry, Universidad Autónoma de Madrid, Calle Francisco Tomás y Valiente, 7, 28049 Madrid, Spain.

[d] Univ Angers, CNRS MOLTECH-ANJOU, SFR MATRIX, F-49000 Angers, France.

[e] Department of Medical Biochemistry and Biophysics, Umeå University, Umeå 90187, Sweden.

[f] Science for Life Laboratory, Umeå University, Umeå, Sweden.

[g] IADCHEM, Institute for Advanced Research in Chemistry, Universidad Autónoma de Madrid, Calle Francisco Tomás y Valiente, 7, 28049 Madrid, Spain.

[h] Departamento de Química Física de Materiales, Instituto de Química Física Blas Cabrera, CSIC 28006, Madrid, Spain. E-mail: lmartinez@iqf.csic.es

1. **Synthesis**

DBI-POE was synthesized and characterized according to our previously reported procedure.[1]

2. **Spectroscopy**
   - **2.1.** Absorption measurements were performed on a JASCO V-730 UV–vis spectrophotometer equipped with a Peltier-type temperature controller (CDF-426S/15). All spectra were recorded in quartz cuvettes with a 10 mm path length.
   - **2.2.** Steady-state fluorescence measurements were carried out on a JASCO FP-8550 spectrofluorometer equipped with a Julabo CD-B5 temperature-control system. All spectra were recorded in quartz cuvettes with a 10 mm path length.
   - **2.3.** Circular dichroism (CD) spectra were recorded on a JASCO J-1500 spectropolarimeter (JASCO Inc., USA) equipped with a Peltier temperature-control unit (CDF-426S/15). The optical chamber was purged with nitrogen before measurement and maintained under a nitrogen atmosphere throughout the experiments. All spectra were recorded in quartz cuvettes with a 10 mm path length.
   - **2.4.** ^{1}H NMR measurements were performed at 298 K using a Bruker 600 or 800 MHz spectrometer.
3. **Aggregation studies**
   - **3.1.** Temperature-dependent optical measurements were carried out in aqueous solution using **DBI-POE** at 10 μM for absorption experiments and 2 μM for emission experiments. Spectra were collected from 10 °C to 100 °C.
4. **ROS generation assays**
   - **4.1.** 9,10-Anthracenediyl-bis(methylene)dimalonic acid (ABDA; MedChemExpress) was used as a chemical probe for singlet oxygen ($^1O_2$)-associated oxidation at a final concentration of 30 μM in 50 mM Tris buffer (pH 6.8) containing 100 mM KCl. Samples were prepared under identical buffer conditions and contained **DBI-POE** (5 μM), either alone or in the presence of *AS1411* or the corresponding control oligonucleotide (5 μM). The samples were irradiated using a Hamamatsu Photonics L9588-04 light source equipped with a 485 ± 20 nm bandpass filter at a power density of 5 mW cm^{-2}, with irradiation times ranging from 0 to 30 min. After each irradiation interval, the absorption spectrum of ABDA was recorded. Sodium azide (NaN_3) was used as a $^1O_2$ quencher at a final concentration of 10 mM. For each measurement, an appropriate reference cuvette

containing the corresponding additives was used to correct for background contributions. Under these irradiation conditions, ABDA self-bleaching was negligible both in the absence and presence of NaN_3. The $^1O_2$ quantum yield (Φ_Δ) of the **DBI-POE**:*AS1411* complex was determined using methylene blue (MB) as a reference photosensitizer, with $\Phi_{\Delta(\mathrm{MB})}$ = 0.52 in water. ABDA bleaching followed first-order decay kinetics, and the corresponding rate constants were obtained by linear regression. The Φ_Δ value was calculated by comparing the ABDA bleaching rate and the integrated absorption of the **DBI-POE** complex with those of MB, according to Eq. 1:

$$\Phi_{\Delta(DBI-POE:AS1411)} = \Phi_{\Delta(MB)} \times \frac{k_{(DBI-POE:AS1411)}}{k_{MB}} \times \frac{\int_{465}^{505} A_{MB}(\lambda)d\lambda}{\int_{465}^{505} A_{(DBI-POE:AS1411)}(\lambda)d\lambda} \quad Eq. 1$$

where $k_{\mathrm{DBI\text{-}POE:AS1411}}$ and k_{MB} are the rate constants for ABDA bleaching in the presence of **DBI-POE**:*AS1411* and MB, respectively, and the integrals represent the integrated absorbance of the MB and **DBI-POE**:*AS1411* over the specified wavelength range.

4.2. Dihydrorhodamine 123 (DHR-123; MedChemExpress) was used as a fluorescence probe for superoxide ($O_2^{\bullet-}$)-associated radical oxidation at a final concentration of 5 µM. Samples were prepared in 50 mM Tris buffer (pH 6.8) containing 100 mM KCl and contained **DBI-POE** (5 µM), either alone or in the presence of *AS1411* or the corresponding control oligonucleotide (5 µM). Samples were irradiated using the same light source, bandpass filter, and power density described above, with irradiation times ranging from 0 to 10 min. After each irradiation interval, DHR-123 fluorescence was recorded using excitation at 488 nm.

4.3. Hydroxyphenyl fluorescein (HPF; MedChemExpress) was used as a fluorescence probe for hydroxyl radicals ($OH^\bullet$) detection at a final concentration of 5 µM. Samples were prepared in 50 mM Tris buffer (pH 6.8) containing 100 mM KCl and contained **DBI-POE** (5 µM), either alone or in the presence of *AS1411* or the corresponding control oligonucleotide (5 µM). Samples were irradiated using the same light source, bandpass filter, and power density previously described, with irradiation times ranging from 0 to 15 min. After each irradiation interval, HPF fluorescence was recorded using excitation at 490 nm.

5. DNA binding studies

5.1. Absorption and fluorescence titration experiments were performed at 25 °C in 50 mM Tris-phosphate buffer, pH 6.8, supplemented with 100 mM KCl. **DBI-POE** was kept at a fixed concentration of 10 μM for absorption measurements and 2 μM for fluorescence measurements, while the DNA structures were gradually added over a concentration range of 0–20 μM. After each addition, the samples were allowed to equilibrate for 5 min before spectral acquisition. For fluorescence measurements, samples were excited at 489 nm and emission spectra were recorded from 495 to 750 nm.

5.2. Fluorescence polarization measurements were performed at room temperature using a VANTAstar plate reader equipped with fluorescence polarization optics and 482 ± 16 nm excitation / 530 ± 40 nm emission filters. Measurements were carried out in black, flat-bottom, low-volume 384-well polystyrene microplates with a non-binding surface treatment (Corning). **DBI-POE** was kept at a constant concentration, while *AS1411* concentration was varied as indicated. Measurements were performed in triplicate. For each replicate, the polarization value of **DBI-POE** alone was subtracted as baseline correction, and the data are reported as mean ± SD. Baseline-corrected fluorescence polarization data were fitted using Bindfit with a 1:1 binding model.

5.3. The binding stoichiometry of the **DBI-POE**:*AS1411* complex was evaluated by Job's plot analysis using fluorescence emission as the readout. Experiments were performed in 50 mM Tris buffer, pH 6.8, supplemented with 100 mM KCl. **DBI-POE** and *AS1411* were mixed at different molar ratios while keeping the total analytical concentration of **DBI-POE** plus *AS1411* constant. After equilibration, samples were excited at 489 nm and fluorescence emission spectra were recorded. The fluorescence change associated with complex formation was plotted as a function of the mole fraction of **DBI-POE**.

5.4. Kinetic fluorescence displacement experiments were performed to evaluate whether PhenDC3 could compete with **DBI-POE** for binding to the *AS1411* aptamer. A preformed 1:1 **DBI-POE** complex was prepared by mixing **DBI-POE** and *AS1411* at final concentrations of 2.0 μM each in 50 mM Tris-phosphate buffer, pH 6.8, supplemented with 100 mM KCl. Two systems were then monitored under identical conditions: the **DBI-POE** complex alone and the **DBI-POE** complex after addition of PhenDC3 at a final

concentration of 2.0 μM. Fluorescence intensity was monitored at 566 nm for 10 min following excitation at 489 nm.

5.5. Cy5 fluorescence-based binding assays were performed in 50 mM Tris buffer, pH 6.8, supplemented with 100 mM KCl. For the *AS1411*-binding assay, *AS1411* labelled with Cy5 at the *3′*-end was used at a final concentration of 50 nM and titrated with increasing concentrations of **DBI-POE** from 0 to 1000 nM. After each addition, Cy5 fluorescence was recorded to monitor **DBI-POE**-induced changes in the fluorescence signal of the labelled aptamer.

For nucleolin-binding experiments, Cy5-labelled *AS1411* was used at a final concentration of 20 nM. Recombinant human nucleolin/NCL protein carrying a C-terminal human IgG1 Fc tag was purchased from ACROBiosystems (Cat. No. NUL-H5253) and used according to the supplier's instructions. Three systems were compared: Cy5-labelled *AS1411* alone, Cy5-labelled *AS1411* preincubated with **DBI-POE** and kept in the dark, and Cy5-labelled *AS1411* preincubated with **DBI-POE** followed by light irradiation. **DBI-POE** was used at a final concentration of 20 nM. Recombinant nucleolin was then titrated from 0 to 50 nM, and Cy5 fluorescence was monitored after each addition. Binding profiles were generated from the nucleolin-dependent changes in Cy5 fluorescence intensity. Data were fitted using Bindfit.

5.6. CD measurements were carried out in 10 mM Tris buffer, pH 6.8, supplemented with 100 mM KCl. *AS1411* or *AT11* was used at a fixed concentration of 2.0 μM and titrated with **DBI-POE**. CD spectra were recorded after sample equilibration to evaluate the effect of **DBI-POE** binding on the *AS1411* or *AT11* G4 fold. Each spectrum represents the average of three accumulations. All spectra were corrected by subtraction of the appropriate reference spectra. Light-irradiation CD experiments were performed under the same buffer conditions using *AS1411* alone, *AS1411*/**DBI-POE**, or *AT11*/**DBI-POE** at a fixed oligonucleotide concentration of 2.0 μM. Samples were irradiated using the same light source, bandpass filter, and power density described above. CD spectra were recorded after increasing irradiation times of 0, 2.5, 5, 10, 20, 30, 40, and 60 min for *AS1411*/**DBI-POE**, with an additional 90 min time point recorded for *AS1411* alone and for *AT11*/**DBI-POE**. CD melting experiments were performed using *AS1411* at 2.0 μM, either alone or in the presence of **DBI-POE** at 2.0 μM, in 10 mM Tris buffer, pH 6.8, supplemented with

100 mM KCl. Where indicated, samples were irradiated prior to melting analysis for 30, 60, or 90 min using the same irradiation setup previously described. Melting profiles were obtained by monitoring temperature-dependent changes in the characteristic *AS1411* CD signal, and melting temperature values were determined by sigmoidal nonlinear fitting of the resulting curves.

5.7. ^{1}H NMR experiments were performed in 10 mM Tris-HCl buffer, pH 6.8, containing 10% D_2O in 70 mM KCl. For the analysis of G4 folding, *AS1411*, the C-rich sequence, and the G-to-C mutant were prepared at a final concentration of 225 µM. For **DBI-POE** titration experiments, *AT11* was used at a fixed concentration of 350 µM and titrated with increasing concentrations of **DBI-POE**. The final **DBI-POE** concentrations were 0, 35, 87.5, 175, 262.5, and 350 µM. For light-irradiation experiments, samples containing *AT11* at 350 µM and **DBI-POE** at 175 µM were irradiated for 0, 5, 10, 20, 40, and 50 min using the same irradiation setup previously described.

6. DNA sample preparation

6.1. Oligonucleotides were purchased from commercial suppliers (Sigma-Aldrich) and used without further modification unless otherwise stated. Stock solutions were prepared by dissolving the oligonucleotides in ultrapure DNase- and RNase-free water and stored at 5 °C. Oligonucleotide concentrations were determined by UV–vis absorption spectroscopy using the molar extinction coefficients at 260 nm listed in Supplementary Table 1, which were calculated with the IDT OligoAnalyzer tool. For fluorophore-labelled oligonucleotides, the concentrations provided by the supplier were used. Prior to use, oligonucleotides were folded by heating at 95 °C for 5 min in the presence of 100 mM KCl, followed by slow cooling to room temperature overnight. A complete list of the oligonucleotides used in this study is provided in Supplementary Table 1.

Supplementary Table 1. List of the oligonucleotides used for biophysical studies.

Name	Sequence (5´-to-3´)	Length (nt)	Extinction Coeff. (M^{-1} cm^{-1})	GC (%)
AS1411	GGTGGTGGTGGTTGTGGTGGTGGTGG	26	250800	65.4
AS1411(Cy5)	GGTGGTGGTGGTTGTGGTGGTGGTGG(Cy5)	26	/	65.4
(Cy5)*AS1411*	(Cy5)GGTGGTGGTGGTTGTGGTGGTGGTGG	26	/	65.4
AT11	TGGTGGTGGTTGTTGTGGTGGTGGTGGT	28	264800	57.1
AS1411 (C-rich)	CCACCACCACCACAACCACCACCACC	26	233800	65.4
AS1411 (G-to-C mut)	GCTGCTGCTGCTTATCGTCGTCGTCG	26	226100	61.2
dsDNA	CAATCGGATCGAATTCGATCCGATTG	26	253200	46.2

7. Primer extension assay

A *TET*-labelled primer was annealed overnight at room temperature to the corresponding DNA template in 10 mM Tris buffer, pH 7.4, containing 50 mM of either LiCl or KCl. The primer was used at 1 μM and the DNA templates at 2 μM. The following templates were used: *AT11*, *AT11*-8oxoG, *AS1411*, *AS1411* (C-rich), and *AS1411* (G-to-C mut). After annealing, the primer–template substrates were diluted to a final DNA concentration of 40 nM in reaction buffer containing 10 mM Tris, 8 mM $MgCl_2$, and either KCl or LiCl, as indicated. Where specified, samples were supplemented with **DBI-POE** at a final concentration of 1 μM and irradiated for 5 or 15 min under a fluorescence microscope equipped with an Olympus BX61 platform, an Olympus U-RFL-T burner, and a U-FBWA FITC filter cube. Irradiation was performed using excitation light in the 460–495 nm range, ensuring that the light beam passed through the entire sample volume. Vehicle-control reactions contained 1% DMSO. After irradiation, Bsu DNA Polymerase I, Large Fragment (New England Biolabs), was added to each sample at a final concentration of 0.05 U/μL. Samples were equilibrated at 37 °C in a water bath for 10 min, and primer extension reactions were initiated by addition of dNTPs to a final concentration of 0.2 mM. Reactions were allowed to proceed for 1 min and then stopped by addition of an equal volume of 20 mM EDTA. Reaction products were purified using the Monarch® PCR & DNA Cleanup Kit (5 μg binding capacity; New England Biolabs). Prior to electrophoresis, samples were denatured by mixing with 95% formamide at a 1:1 ratio, heating at 95 °C for 5 min, and snap-cooling on ice. Denatured samples were loaded onto freshly prepared denaturing sequencing gels composed of 10% polyacrylamide, 8 M urea, 25% formamide, and 1× TBE buffer. Gels were run at 60 W for 1 h 45 min. Gel images were acquired using a Typhoon Scanner FLA 9500 (GE Healthcare) with the Alexa 532 fluorescence channel. Band intensities were analyzed in Image Lab 6.1.0 software (Bio-Rad), and the signal from each band was calculated as a proportion percentage of the total fluorescence detected within the corresponding lane.

<u>Primer:</u>

5′- *TET*-TGAAAACATTATTAATGGCGTCGAGCGTCCG-3′

<u>*AS1411* template:</u>

5′-**GGTGGTGGTGGTTGTGGTGGTGGTGG**atatatatatCGGACGCTCGACGCCATTAATAATGTTTTCA-3′

AT11 template:

5′-**TGGTGGTGGTTGTTGTGGTGGTGGTGGT**atatatatatCGGACGCTCGACGCCATTAATAATGTTTTCA-3′

AT11-8-oxoG template:

5′-**TGGTGGTGGTTGTTGTGG*TGG*TGG*TGGT**atatatatatCGGACGCTCGACGCCATTAATAATGTTTTCA-3′

* = 8-oxoG

AS1411 (C-rich) template:

5′-**CCACCACCACCACAACCACCACCACC**atatatatatCGGACGCTCGACGCCATTAATAATGTTTTCA-3′

AS1411 (G-to-C mut) template:

5′-**GCTGCTGCTGCTTATCGTCGTCGTCG**atatatatatCGGACGCTCGACGCCATTAATAATGTTTTCA-3′

8. Dot blotting assay

AS1411 and the *HIF-1α* (positive control) G4-forming oligonucleotide, previously resuspended in nuclease-free water, were diluted in folding buffer composed of 10 mM Tris, pH 7.4, and 100 mM KCl to a final concentration of 8 μM. The oligonucleotides were folded isothermally at room temperature overnight. Where indicated, samples were supplemented with **DBI-POE** at a 1:1 molar ratio with *AS1411* or *HIF-1α* and irradiated for 20 min under a fluorescence microscope equipped with an Olympus BX61 platform, an Olympus U-RFL-T burner, and a U-FBWA FITC filter cube. Irradiation was performed using excitation light in the 460–495 nm range. Control samples contained 8% DMSO. Following irradiation, DNA samples were purified using the Monarch® PCR & DNA Cleanup Kit (New England Biolabs) and resuspended in folding buffer to obtain 2 μM DNA solutions. Dot blot analysis was performed according to a previously reported protocol with minor modifications.[1] Samples were loaded onto an Amersham Hybond-N$^+$ nylon membrane (Cytiva) using a Bio-Dot microfiltration apparatus (Bio-Rad), following the manufacturer's instructions. The membrane was blocked for 1 h at room temperature in 5% (w/v) milk prepared in TBS buffer containing 20 mM Tris, pH 7.4, and 150 mM KCl, and then washed for 10 min with TBST buffer containing 20 mM Tris, pH 7.4, 150 mM KCl, and 0.1% (v/v) Tween-20. The membrane was then incubated overnight at 4 °C with BG4 antibody at 0.1 μg/mL, purified according to Lyu et al.[2], and diluted in 1% (w/v) milk in TBS. After washing, the membrane was incubated with mouse anti-FLAG antibody (Novus Biologicals, #NBP1-97410) at a 1:5000 dilution for 2 h at room temperature, followed by incubation with HRP-conjugated anti-mouse IgG

antibody (R&D Systems, #HAF007) at a 1:5000 dilution for 1 h at room temperature. Each antibody incubation step was followed by three washes with TBST. Chemiluminescent signal was developed using Radiance Q Chemiluminescent Substrate (Azure Biosystems, #AC2101), and images were acquired using an Azure Biosystems c280 imaging system.

The G4-forming DNA sequences used in this assay were:

HIF-1α: 5′-GCGCGGGGAGGGGAGAGGGGGCGGGAGCGCG-3′

AS1411: 5′-GGTGGTGGTGGTTGTGGTGGTGGTGG-3′

9. Native PAGE

AT11 and *AS1411* G4 oligonucleotides were dissolved in water and diluted to a final concentration of 2 µM in folding buffer containing 10 mM Tris-HCl and 100 mM KCl. Samples were allowed to fold overnight at room temperature. Where indicated, samples were irradiated with blue light for 5 or 15 min using a 40 W COB LED lamp (Fun Generation LED Pot COB 40W RGB WW, Germany). Sample tubes were positioned centrally relative to the light source to ensure consistent irradiation conditions. Before electrophoresis, 50% glycerol was added to the samples at a 1:4 (v/v) glycerol-to-sample ratio. Samples were then loaded onto 15% native polyacrylamide gels prepared in 1× TBE containing 10 mM KCl. Electrophoresis was performed at 70 V for 80 min using 1× TBE supplemented with 10 mM KCl as the running buffer. Following electrophoresis, gels were stained for 10 min with 5 µM Thiazole Orange prepared in running buffer and subsequently washed three times with running buffer. Fluorescent bands were visualized using a ChemiDoc MP Imaging System (Bio-Rad) with the Alexa 488 detection channel.

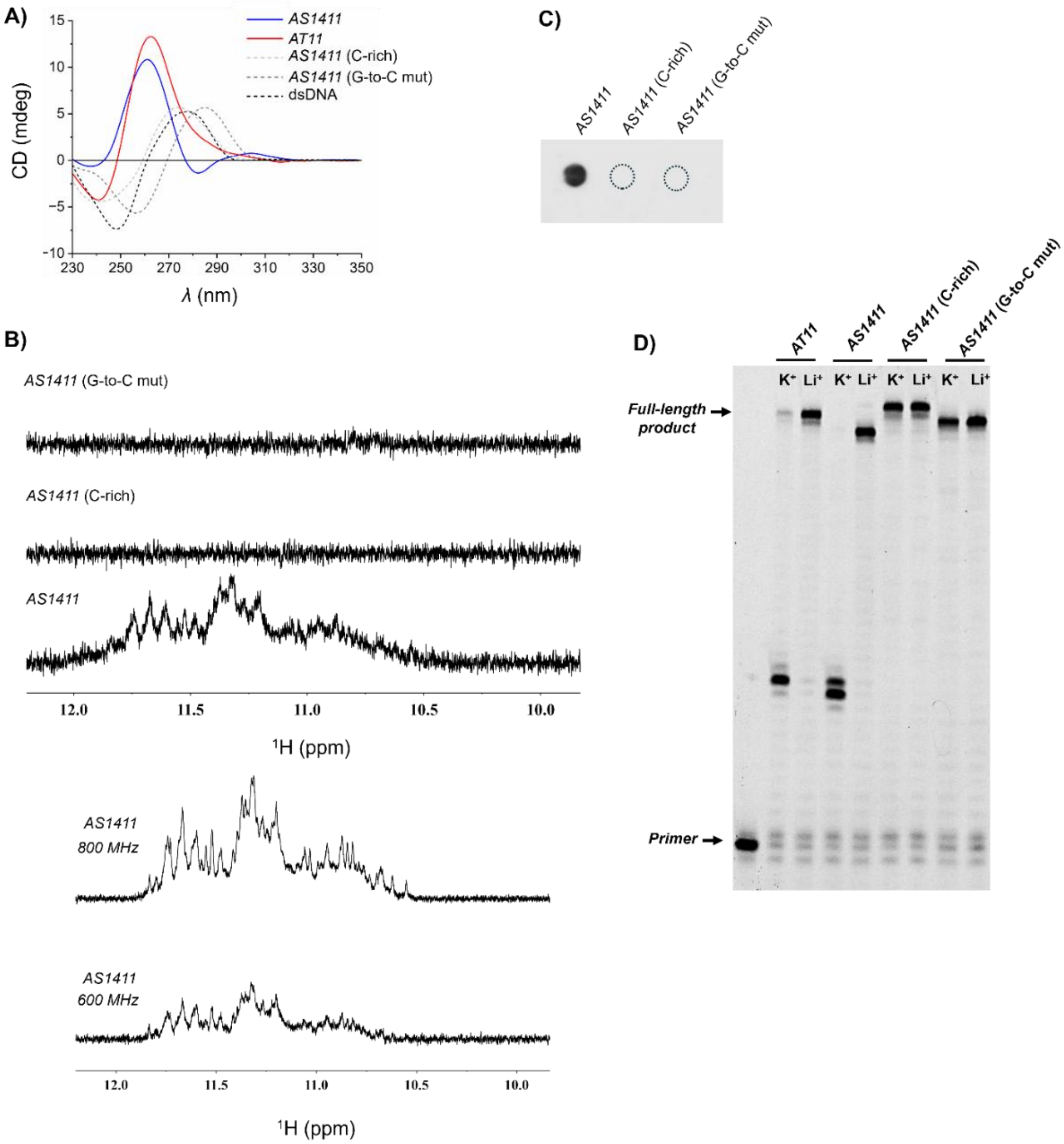

Figure S1. Orthogonal validation of G4 formation. **A)** CD spectra of *AS1411*, *AT11*, *AS1411* (C-rich), *AS1411* (G-to-C mut), and dsDNA. Measurements were performed using DNA sequences at 2 µM in 10 mM Tris buffer (pH 6.8) containing 100 mM KCl. **B)** Top: ^{1}H NMR spectra (600 MHz) of the guanine imino-proton region of *AS1411*, *AS1411* (C-rich), and *AS1411* (G-to-C mut), recorded to assess the formation of hydrogen-bonded G-tetrads. Oligonucleotides were measured at 225 µM in 10 mM Tris buffer (pH 6.8) containing 70 mM KCl. Bottom: ^{1}H NMR spectra (600 and 800 MHz) of the guanine imino-proton region of *AS1411*, recorded at a higher oligonucleotide concentration of 350 µM in 10 mM Tris buffer (pH 6.8) containing 70 mM KCl. **C)** BG4-based dot blot analysis of *AS1411*, *AS1411* (C-rich), and *AS1411* (G-to-C mut) sequences, used to evaluate formation of BG4-recognizable folded G4 structures. Experiments were performed in 10 mM Tris buffer (pH 7.4) containing 100 mM KCl. **D)** Representative primer-extension assay performed using *AT11*, *AS1411*, *AS1411* (C-rich), and *AS1411* (G-to-C mut) templates under G4-stabilizing conditions in 50 mM KCl or non-G4-stabilizing conditions in 50 mM LiCl.

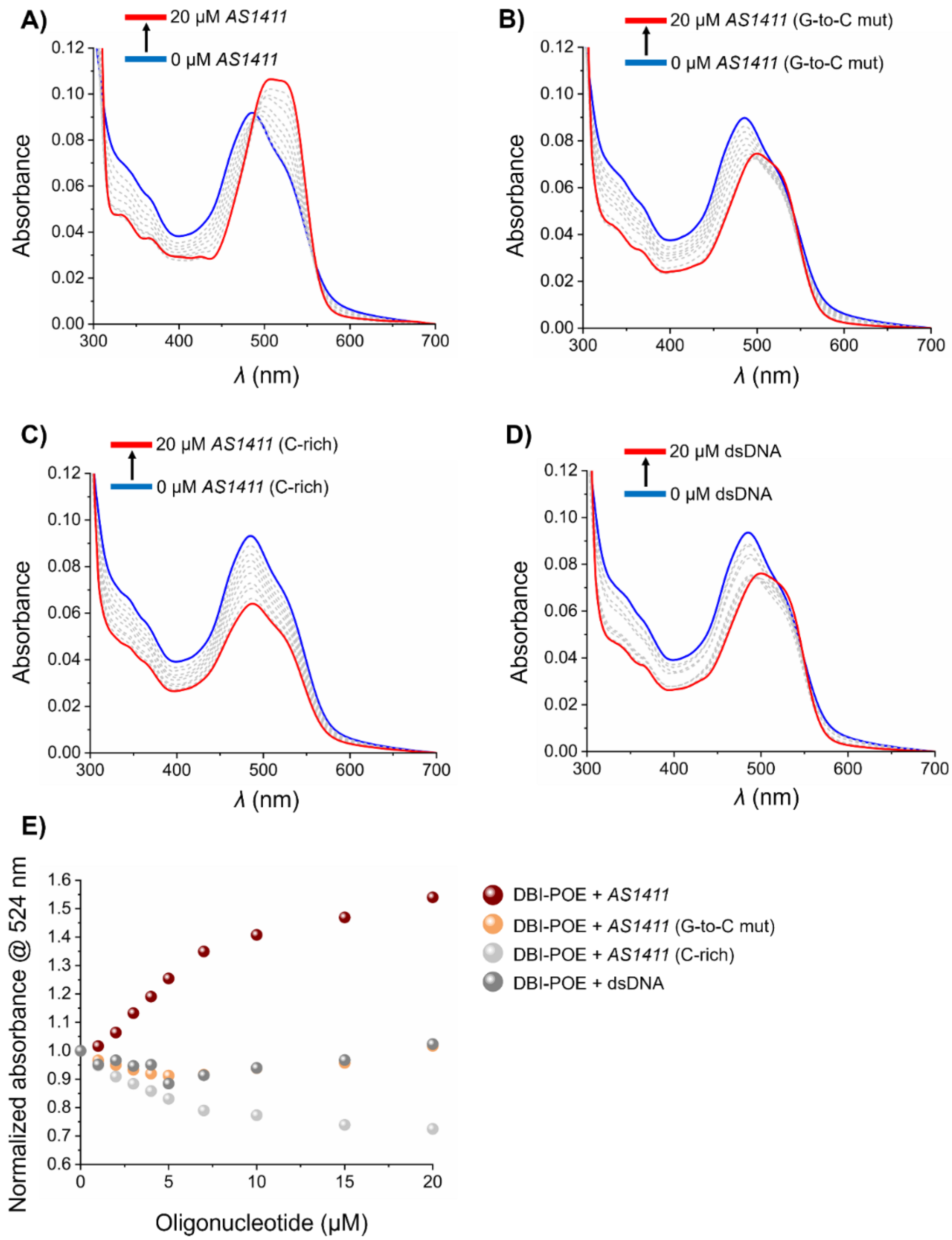

Figure S2. UV–vis absorption response of **DBI-POE** to *AS1411* and non-G4 control sequences. **A–D)** UV–vis absorption spectra of **DBI-POE** recorded upon titration with *AS1411*, *AS1411* (G-to-C mut), *AS1411* (C-rich) and dsDNA to compare G4-dependent and nonspecific nucleic-acid-associated optical changes. Spectra of **DBI-POE** alone are shown in blue, whereas spectra recorded upon increasing oligonucleotide concentration are shown from gray to red. Measurements were performed using **DBI-POE** (10 µM) in 50 mM Tris buffer (pH 6.8) containing 100 mM KCl. Oligonucleotides were titrated from 0 to 20 µM. **E)** Optical response of **DBI-POE** upon oligonucleotide titration, monitored at 524 nm and plotted as a function of oligonucleotide concentration. Data were extracted from the UV–vis absorption spectra shown in panels A–D.

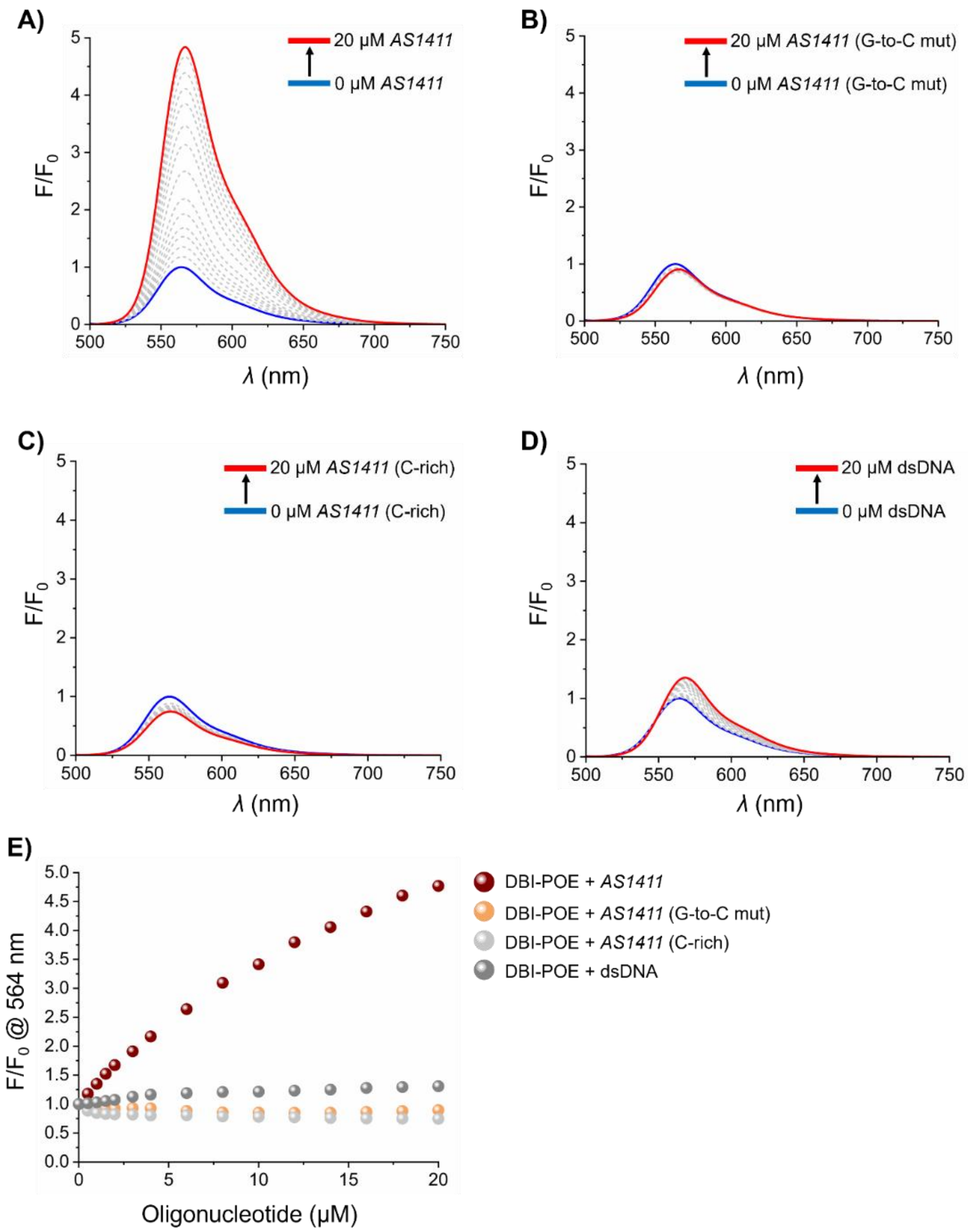

Figure S3. Fluorescence emission response of **DBI-POE** to *AS1411* and non-G4 control sequences. **A–D)** Fluorescence emission spectra of **DBI-POE** recorded upon titration with *AS1411*, *AS1411* (G-to-C mut), *AS1411* (C-rich) and dsDNA to compare G4-dependent and nonspecific nucleic-acid-associated optical responses. Spectra of **DBI-POE** alone are shown in blue, whereas spectra recorded upon increasing oligonucleotide concentration are shown from gray to red. Measurements were performed using **DBI-POE** (2 µM) in 50 mM Tris buffer (pH 6.8) containing 100 mM KCl. Oligonucleotides were titrated from 0 to 20 µM; λ_{exc} = 489 nm. **E)** Fluorescence response of **DBI-POE** upon oligonucleotide titration, monitored at 564 nm and plotted as a function of oligonucleotide concentration. Data were extracted from the fluorescence emission spectra shown in panels A–D.

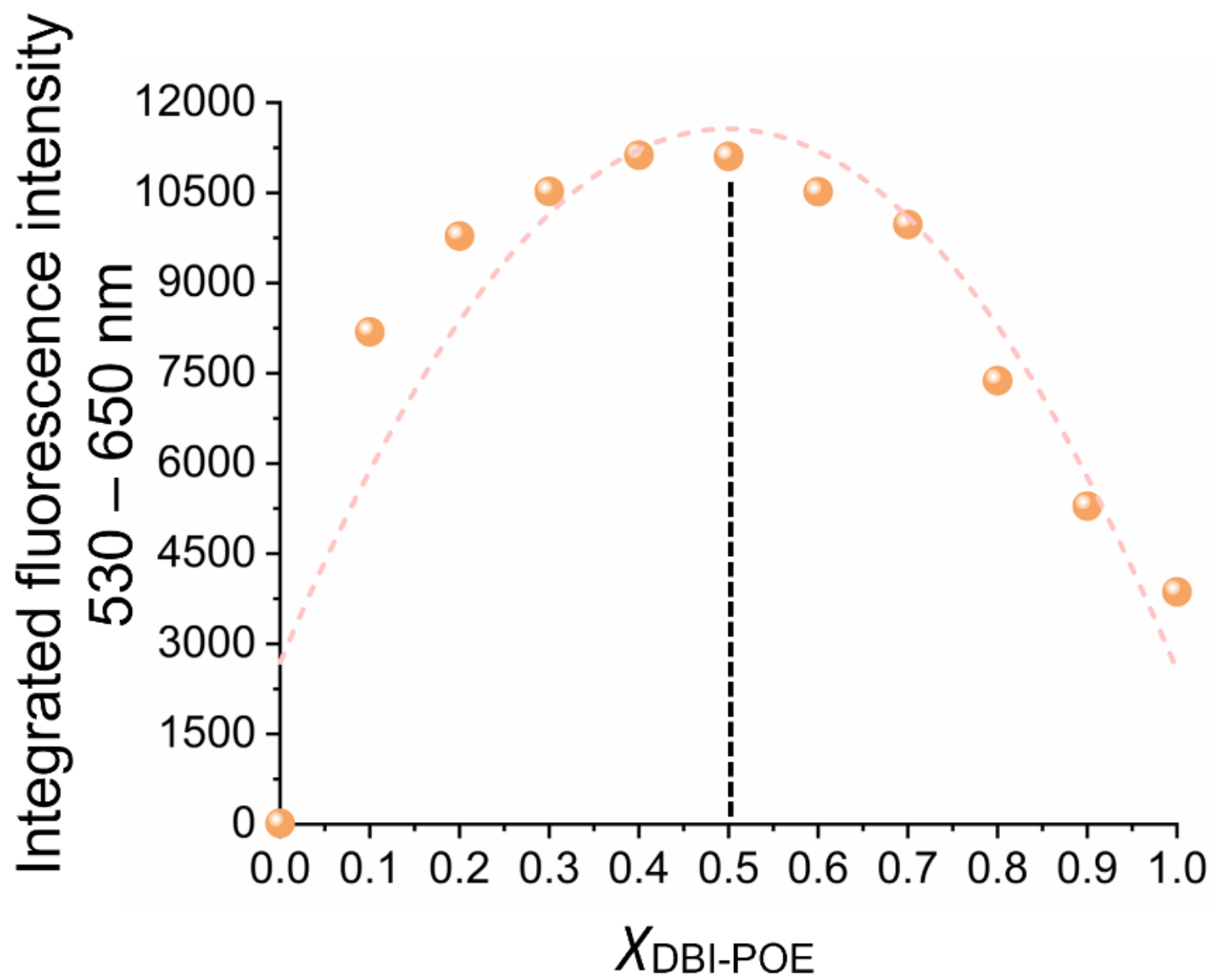

Figure S4. Job's plot analysis of **DBI-POE** binding to *AS1411*. Stoichiometry of the **DBI-POE**/*AS1411* complex was assessed from fluorescence emission changes in 50 mM Tris buffer (pH 6.8) containing 100 mM KCl. The vertical dotted line indicates a 1:1 binding stoichiometry. λ_{exc} = 489 nm.

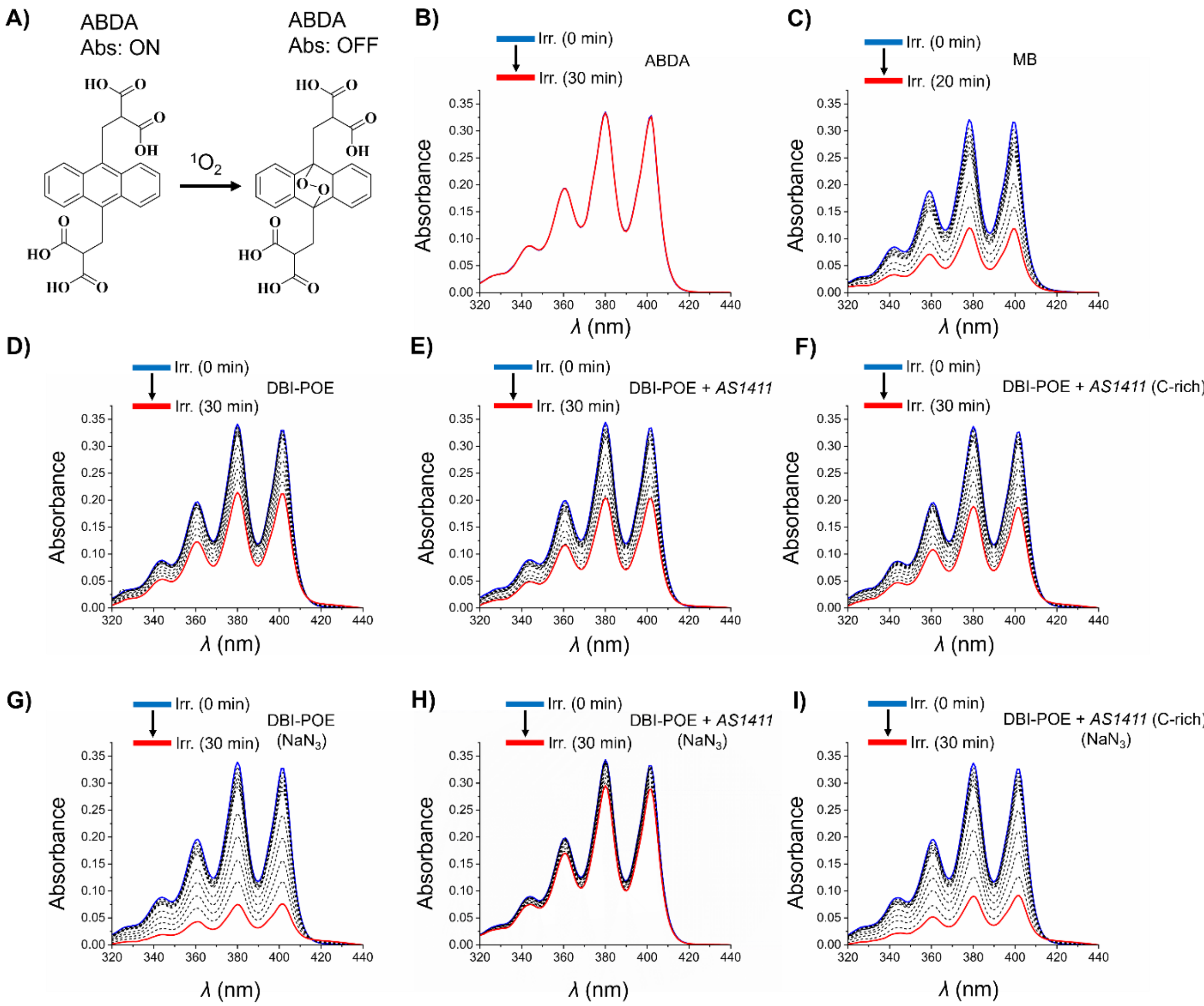

Figure S5. ABDA-based $^1O_2$ assay. **A)** Schematic representation of the ABDA assay. ABDA undergoes oxidative conversion upon reaction with $^1O_2$, leading to a decrease in its characteristic absorption bands. **B)** Control experiment showing the photostability of ABDA under irradiation in the absence of photosensitizer. **C)** Positive-control ABDA bleaching experiment using methylene blue (MB) as a reference $^1O_2$ photosensitizer. Spectra were recorded at different irradiation times. **D–F)** ABDA bleaching experiments performed in the presence of **DBI-POE** alone (D), **DBI-POE** with *AS1411* (E), and **DBI-POE** with *AS1411* (C-rich) (F). **G–I)** ABDA bleaching experiments performed under the same conditions as in panels D–F but in the presence of NaN_3, using **DBI-POE** alone (G), **DBI-POE** with *AS1411* (H), and **DBI-POE** with *AS1411* (C-rich) (I). ABDA = 30 µM, **DBI-POE** = 5 µM, *AS1411* or *AS1411* (C-rich) = 5 µM, and NaN_3 = 10 mM where indicated, in 50 mM Tris buffer (pH 6.8) containing 100 mM KCl. Irradiation was performed at 485 ± 20 nm. Blue spectra correspond to non-irradiated samples, red spectra to the final irradiation time, and black dashed spectra to intermediate irradiation time points.

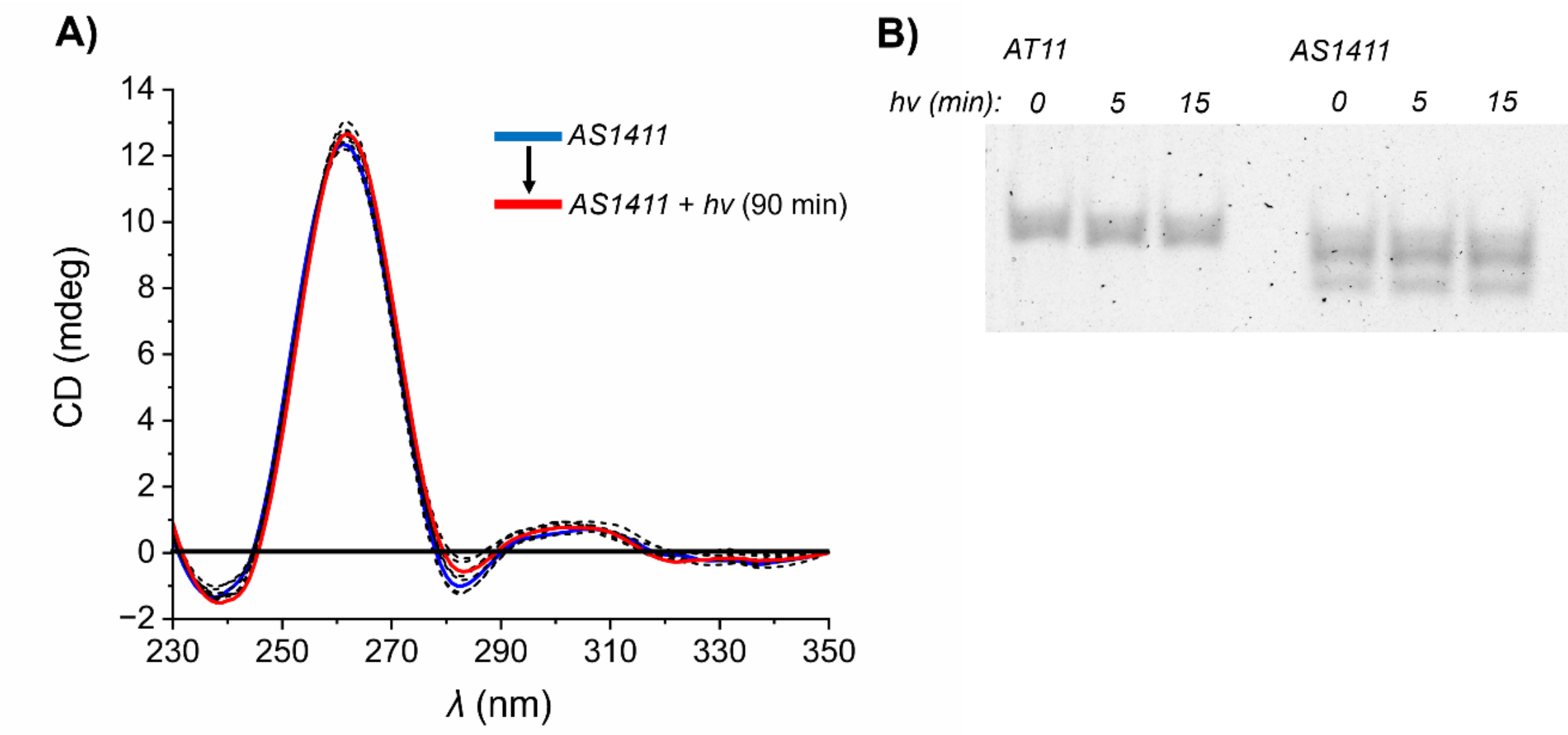

Figure S6. A) CD spectra of *AS1411* recorded after different irradiation times from 0 to 90 min. Measurements were performed using *AS1411* (2 μM) in 10 mM Tris buffer (pH 6.8) containing 100 mM KCl. **B)** Native PAGE analysis of *AT11* and *AS1411* before and after light irradiation for 5 or 15 min. *AT11* predominantly migrated as a single band, consistent with the formation of a single major G4 conformation, whereas *AS1411* displayed multiple bands, consistent with its well-established conformational heterogeneity.

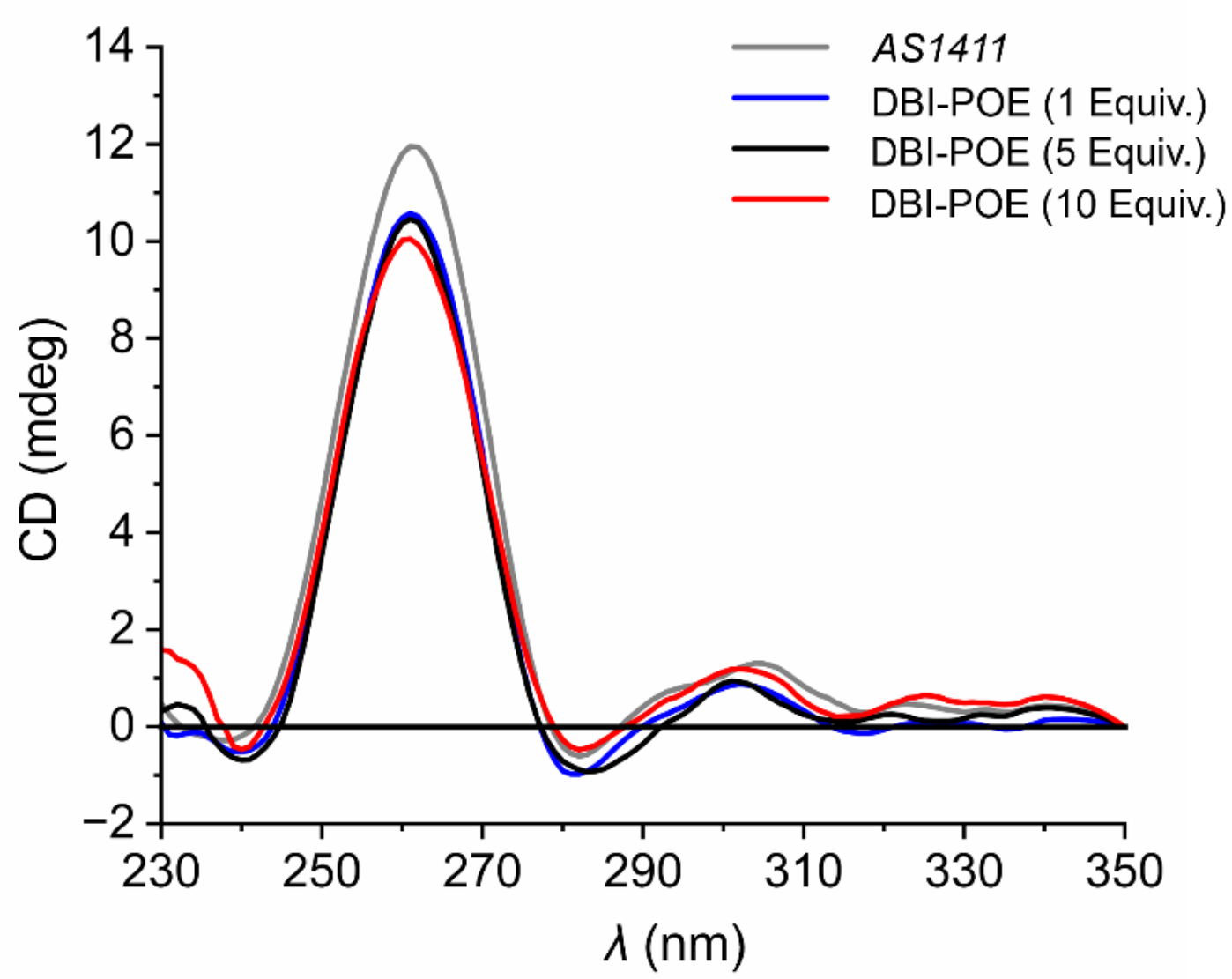

Figure S7. CD spectra of *AS1411* recorded in the absence and presence of increasing concentrations of **DBI-POE**. Measurements were performed using *AS1411* (2 μM) and **DBI-POE** (0–20 μM) in 10 mM Tris buffer (pH 6.8) containing 100 mM KCl.

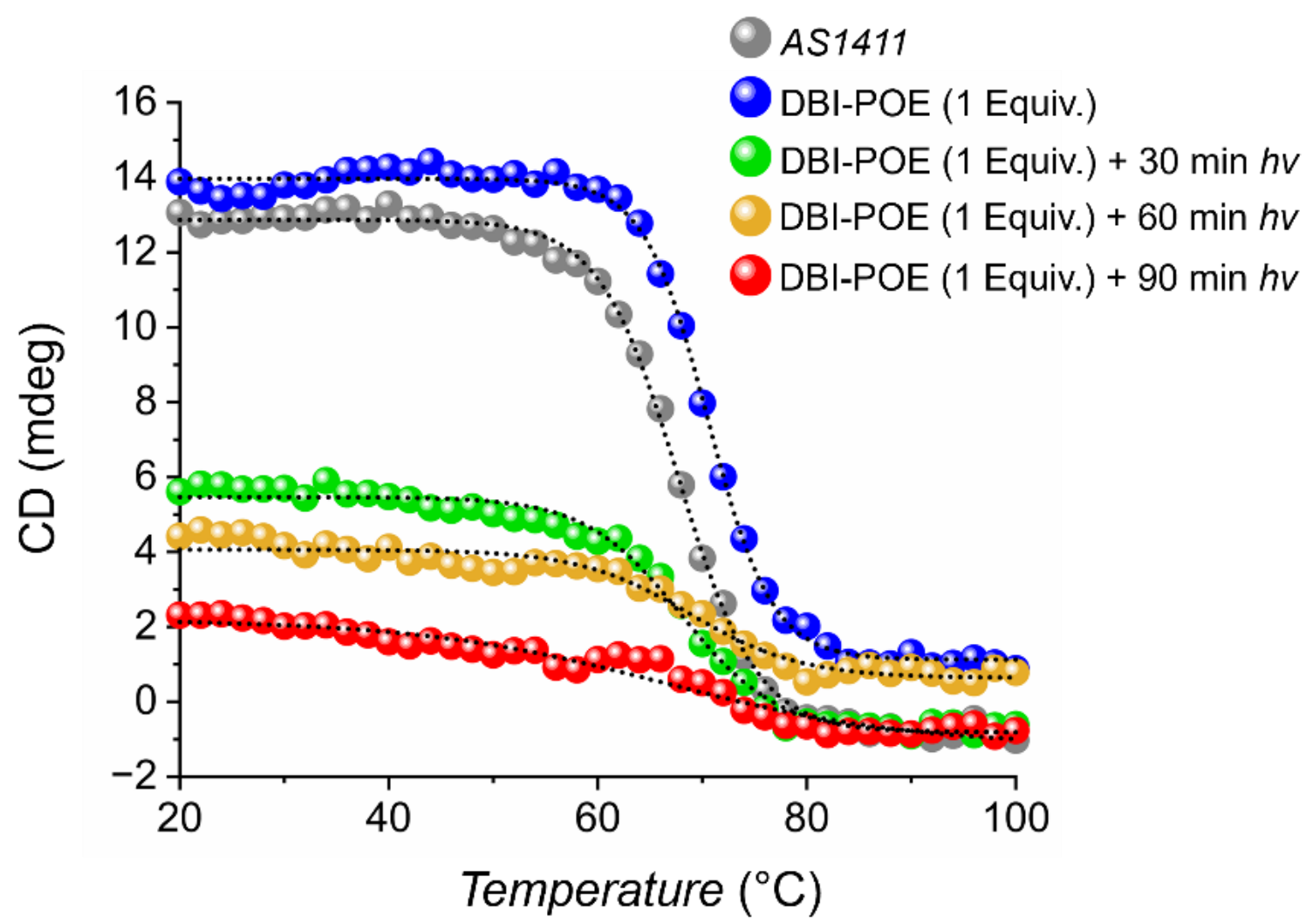

Figure S8. CD-monitored melting profiles of *AS1411* recorded in the absence and presence of **DBI-POE** under different irradiation conditions, plotted at the CD maximum at 262 nm. Measurements were performed using *AS1411* (2 µM) and **DBI-POE** (0 or 2 µM) in 10 mM Tris buffer (pH 6.8) containing 100 mM KCl.

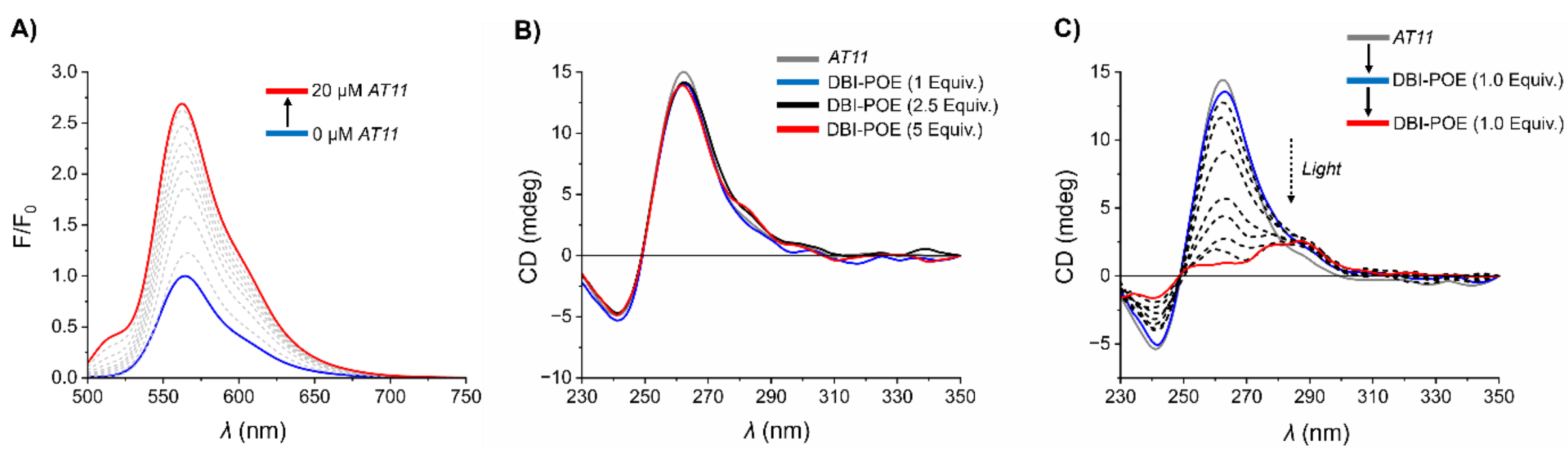

Figure S9. A) Fluorescence emission spectra of **DBI-POE** recorded upon titration with *AT11*. The spectrum of **DBI-POE** alone is shown in blue, whereas spectra recorded upon increasing oligonucleotide concentrations are shown from gray to red. Measurements were performed using **DBI-POE** (2 µM) in 50 mM Tris buffer (pH 6.8) containing 100 mM KCl. *AT11* was titrated from 0 to 20 µM; λ_{exc} = 489 nm. **B)** CD spectra of *AT11* recorded in the absence and presence of increasing concentrations of **DBI-POE**. Measurements were performed using *AT11* (2 µM) and **DBI-POE** (0–10 µM) in 10 mM Tris buffer (pH 6.8) containing 100 mM KCl. **C)** CD spectra of *AT11* recorded in the absence and presence of **DBI-POE** after different irradiation times from 0 to 90 min. Measurements were performed using *AT11* (2 µM) and **DBI-POE** (2 µM) in 10 mM Tris buffer (pH 6.8) containing 100 mM KCl.

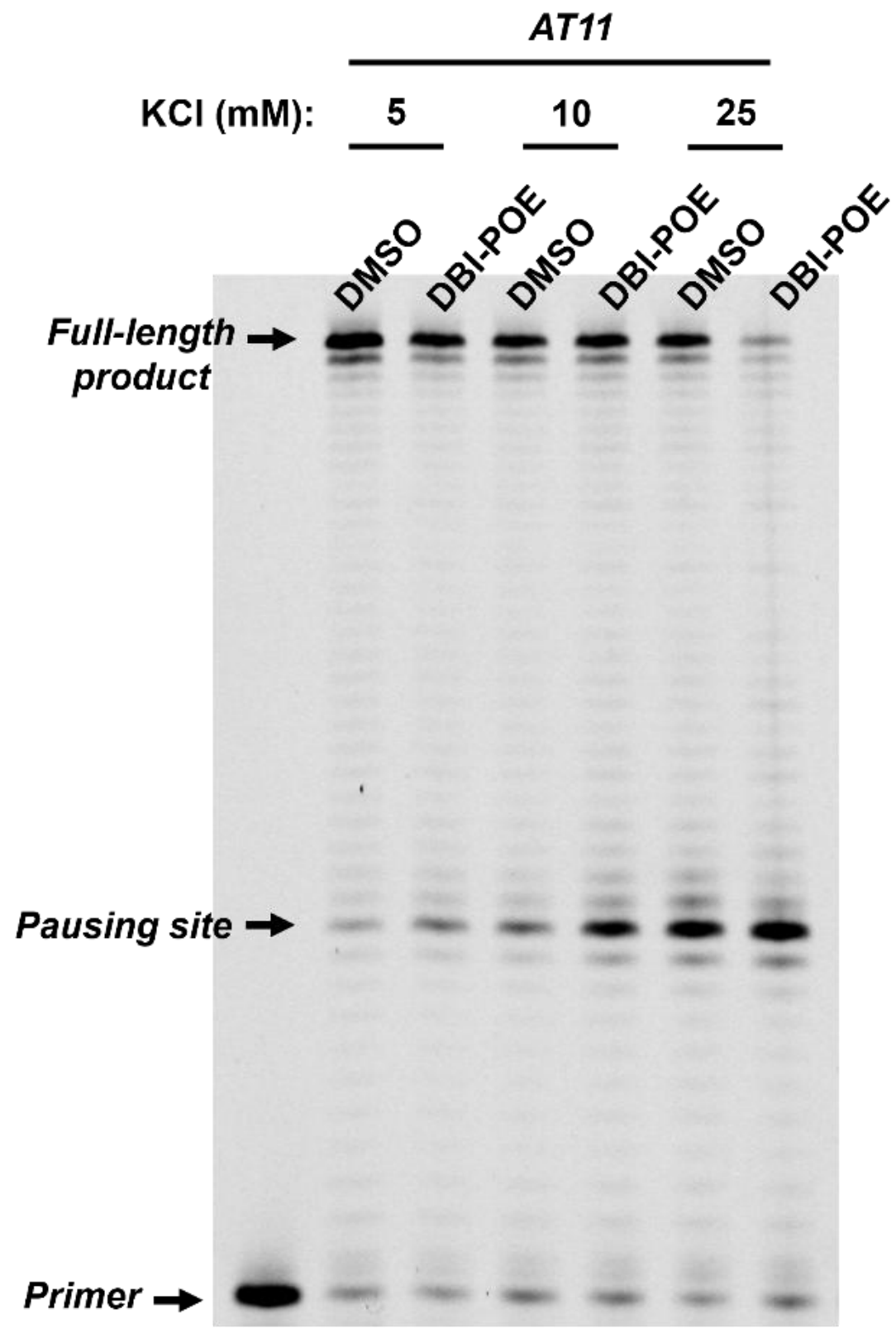

Figure S10. Representative primer-extension assay performed using the *AT11* template under G4-stabilizing conditions in 5, 10, or 25 mM KCl, in the absence or presence of **DBI-POE** (1 μM). Black arrows indicate the non-extended primer, polymerase pausing site, and full-length extension products.

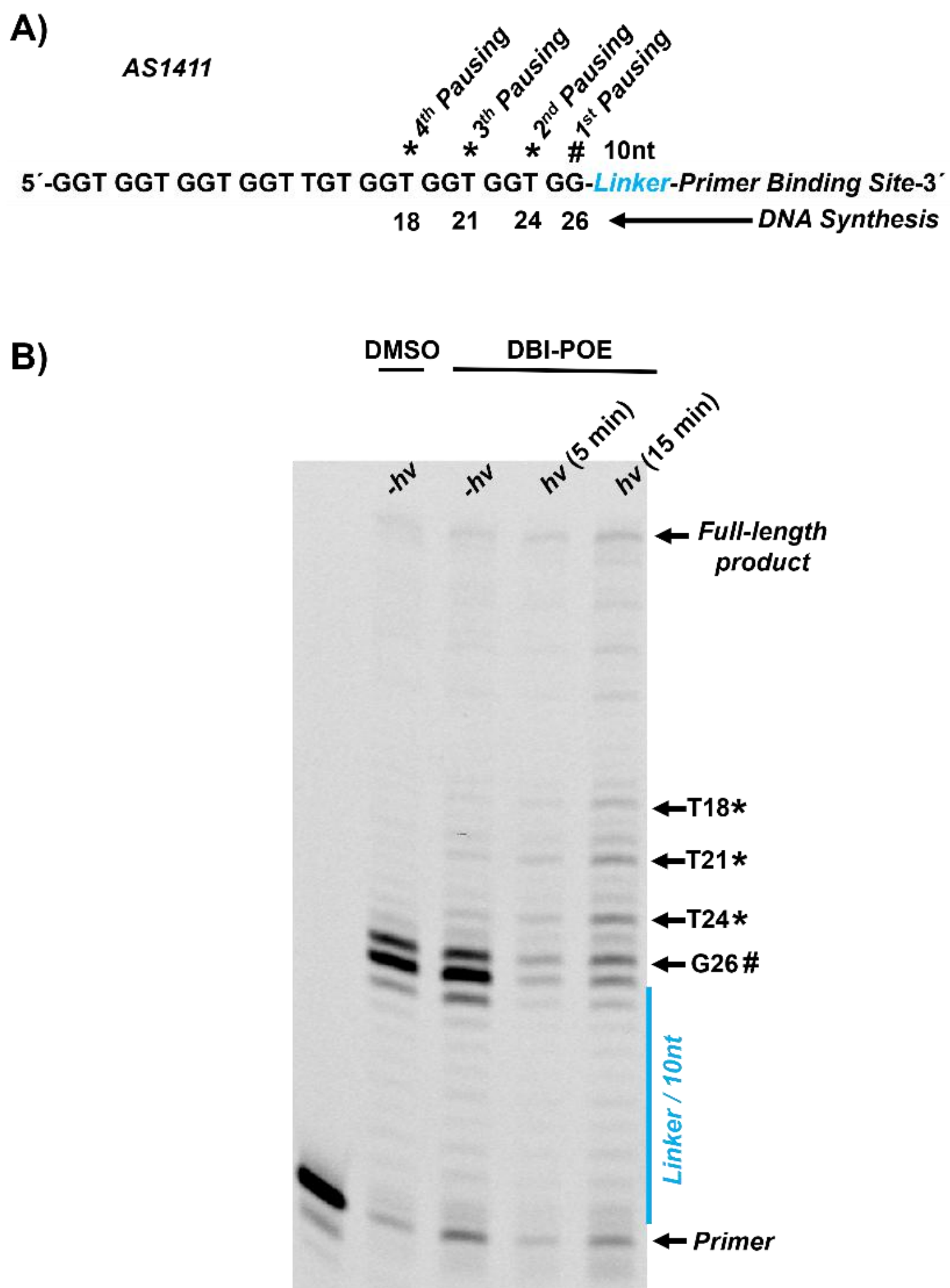

Figure S11. A) Sequence of the AS*1411* template used for primer-extension analysis. Symbols indicate the first, second, third, and fourth polymerase pausing sites detected in panel B. **B)** Representative primer-extension assay performed with the *AS1411* template under G4-stabilizing conditions in 10 mM KCl. Reactions were performed using **DBI-POE** (1 µM). Black arrows indicate the non-extended primer, polymerase pausing sites, and full-length extension products.

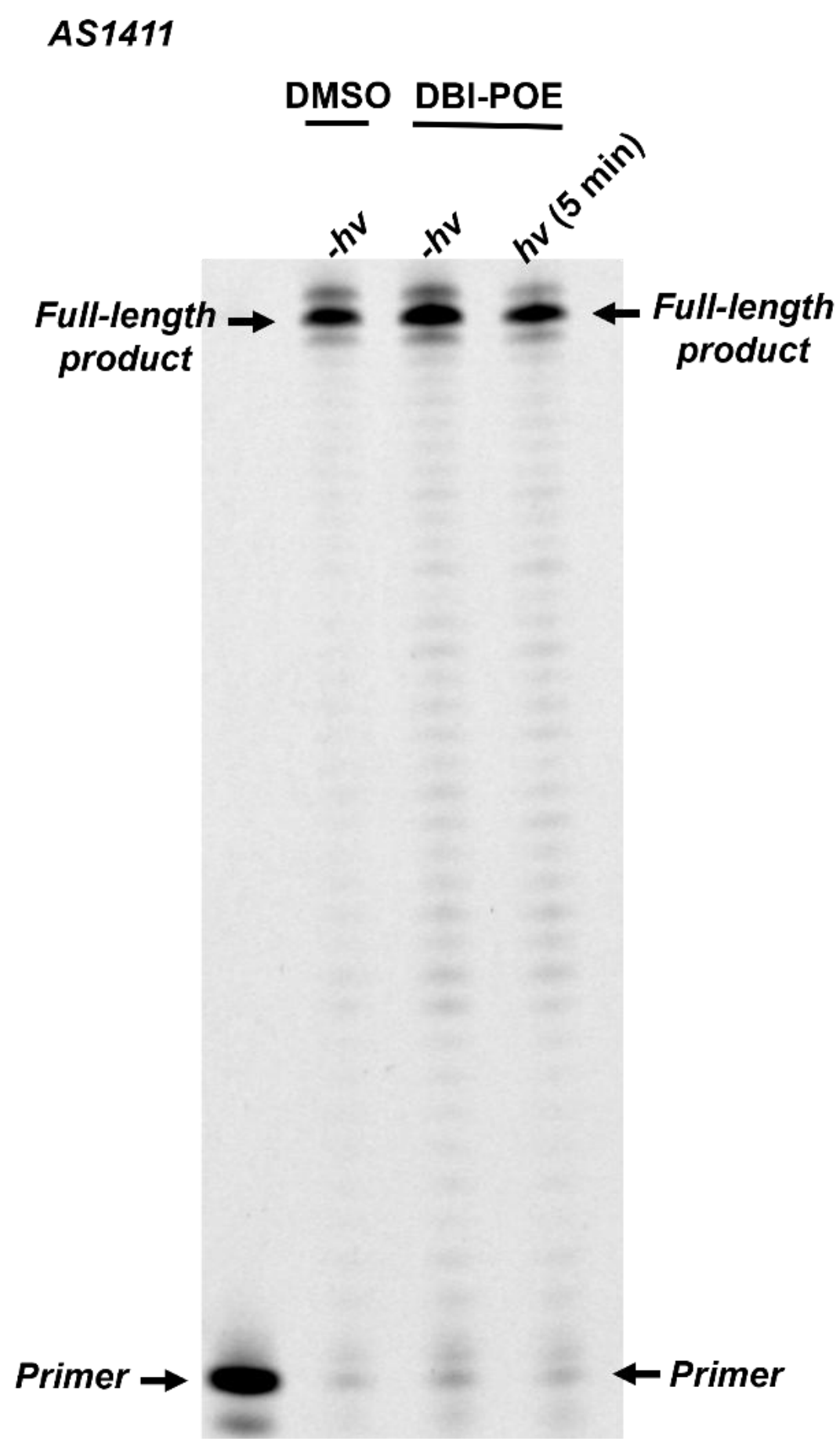

Figure S12. Representative primer-extension assay performed with the *AS1411* template under non-G4-stabilizing conditions in 50 mM LiCl. Reactions were performed using **DBI-POE** (1 μM). Black arrows indicate the non-extended primer and full-length extension products.

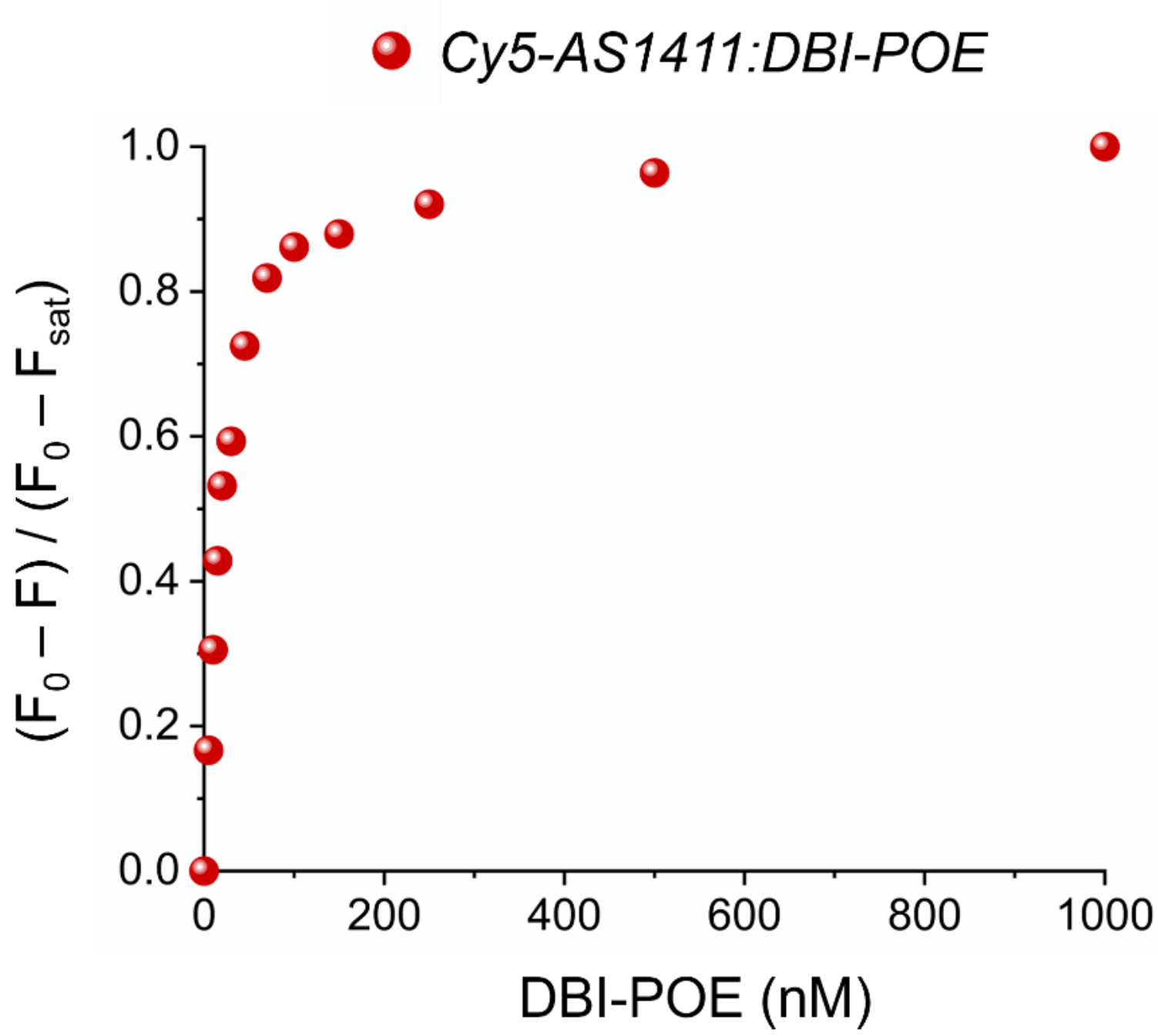

Figure S13. Cy5 fluorescence-based *AS1411*-binding assay. Experiments were performed using *AS1411* labeled with Cy5 at the *3′*-end (50 nM) and **DBI-POE** titrated from 0 to 1000 nM in 50 mM Tris buffer (pH 6.8) containing 100 mM KCl at λ_{exc} = 630 nm. Binding was monitored through **DBI-POE**-induced quenching of Cy5 fluorescence, and the plotted y-axis represents the calculated fraction bound rather than raw fluorescence intensity.

10. Computational details and results

10.1. Classical calculations

To find the binding pockets of **DBI-POE** in the *AS1411* aptamer, docking calculations were performed to identify potential binding sites. Aiming to investigate the intercalation of **DBI-POE**, the *AS1411* aptamer structure, initially taken from the Protein Data Bank (PDB)[3] PDB ID: 2N3M[4], was opened through a combination of molecular dynamics (MD) and umbrella sampling (US) techniques as previously explained.[5] Then, the representative structures of the final opened *AS1411* aptamer structure, which were obtained by a clustering analysis conducted by the cpptraj module of AmberTools20, were used for the docking calculations. Following this procedure, three opened structures to study intercalation were obtained: one in which the two central tetrads were separated (central intercalation), another in which the *3′* tetrad and its closets central tetrad were separated (*3′* intercalation), and a last one in which the *5′* tetrad and its closets central tetrad were separated

(*5′* intercalation). These provided potential binding modes for **DBI-POE**, for which additional MD simulations were performed to study their stability.

To study the stability of the oxidized *AS1411* aptamer, G18, G21 and G24 were substituted by 8-oxoG. Assuming that the ligand leaves its binding site after oxidizing the G4, the MD simulations were performed without **DBI-POE**. In the following, the computational details of all these steps will be explained, except for the US calculations, as we used the same procedure as previously reported.[5, 6]

Docking calculations

The docking calculations were performed with DOCK 6[7] using the same parameters as reported in[7], except for the Hawkins GB/SA rescoring, which was not performed, and the calculation of the ligand charges, which were computed quantum mechanically. Specifically, **DBI-POE** was built following the same procedure as previously reported.[1] Regarding the receptor, the *AS1411* aptamer PDB ID: 2N3M structure was employed to identify the external binding pockets, while the intercalative binding pockets were calculated from the previous US opened central, *5′* and *3′* intercalation structures.

1000 docking calculations were performed for each system. Each one of them rendered 5 different geometries, resulting in a total of 5000 docking positions per system. Based on previously published data[1, 5, 6], we assume that this number of geometries is enough to obtain converged results. Then, we combined the 5000 docking positions in one single trajectory per system. The resulting trajectory was analyzed with cpptraj[8] to get the 10 most representative structures of the docking calculations, i.e., the binding modes that represent the most common docking positions.

Figure S14 shows the representation of the most probable poses for each of the different positions. For the central and *5′* intercalative poses, we selected the most abundant pockets in which the aromatic rings were in the grooves (pockets 0), and the most abundant pockets in which the aromatic rings were stacked in between the tetrads (central →pocket 5, *5′*→pocket 1). In the case of the intercalated *3′*, we selected two different orientations in which the aromatic rings were stacked in between the tetrads (pockets 4 and 6), as the rest of the positions were external. Finally, for the external binding, we selected the two most abundant poses: pockets 0 and 1.

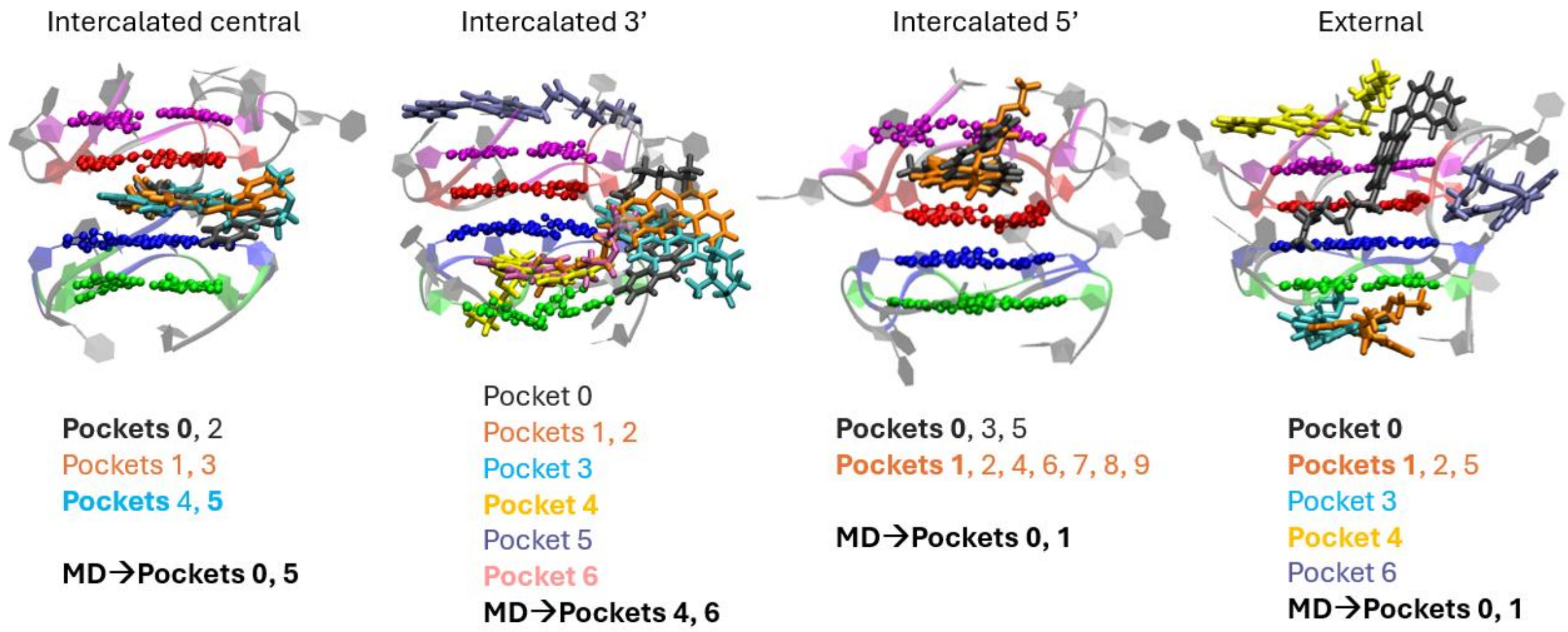

Figure S14. Representation of the most probable poses obtained during the docking for the intercalated and external positions.

Molecular Dynamics simulations

To investigate their stability, the representative structures of the selected pockets were treated with the *tleap* module included in AmberTools20 to prepare the necessary input files to run MD simulations with AMBER20. The Generalised Amber Force Field 2 (GAFF2)[9] was employed to model **DBI-POE**, except for the electrostatic potential charges, that were the previously calculated RESP charges. The remaining force field parameters and *tleap* options were the same as in the US system preparation in previously published articles.[5, 6]

Following the same minimization as in the US simulation, the system was heated from 0 K to 300 K for 1 ns in the NVT ensemble with a 10 kcal·mol^{-1}Å^{-2} force constant restriction to the motion of the Gs in the tetrads and the ligand. Then, the system was equilibrated for 100 ps in the NVT ensemble and 80 ns in the NPT ensemble with the same restrictions. Following NPT calculations were run in which the restrictions were gradually removed in 4 steps of 80 ns each, with force constants of 5, 2.5, 1 and 0.5 kcal·mol^{-1}Å^{-2}. An additional 80 ns NPT simulation was run with a 0.5 kcal·mol^{-1}Å^{-2} restriction on the tetrad guanines and no restrictions on the ligand motion. Finally, a 2 μs NPT production was calculated. The remaining parameters were the same as in the equilibration preceding the US opening in[5, 6].

In most cases, we observe that the G4 structure remains stable during the MD simulation (both intercalated central (Figure S15), intercalated *5′* (Figure S17) and external (Figure S18)) whereas for the intercalated *3′* simulations, the *3′* tetrad structure seems less stable (Figure S16). Regarding the stability of the ligand, it remains in the same position in all simulations except for the external pocket 0, where it moves to the *5′* tetrad (Figure S18).

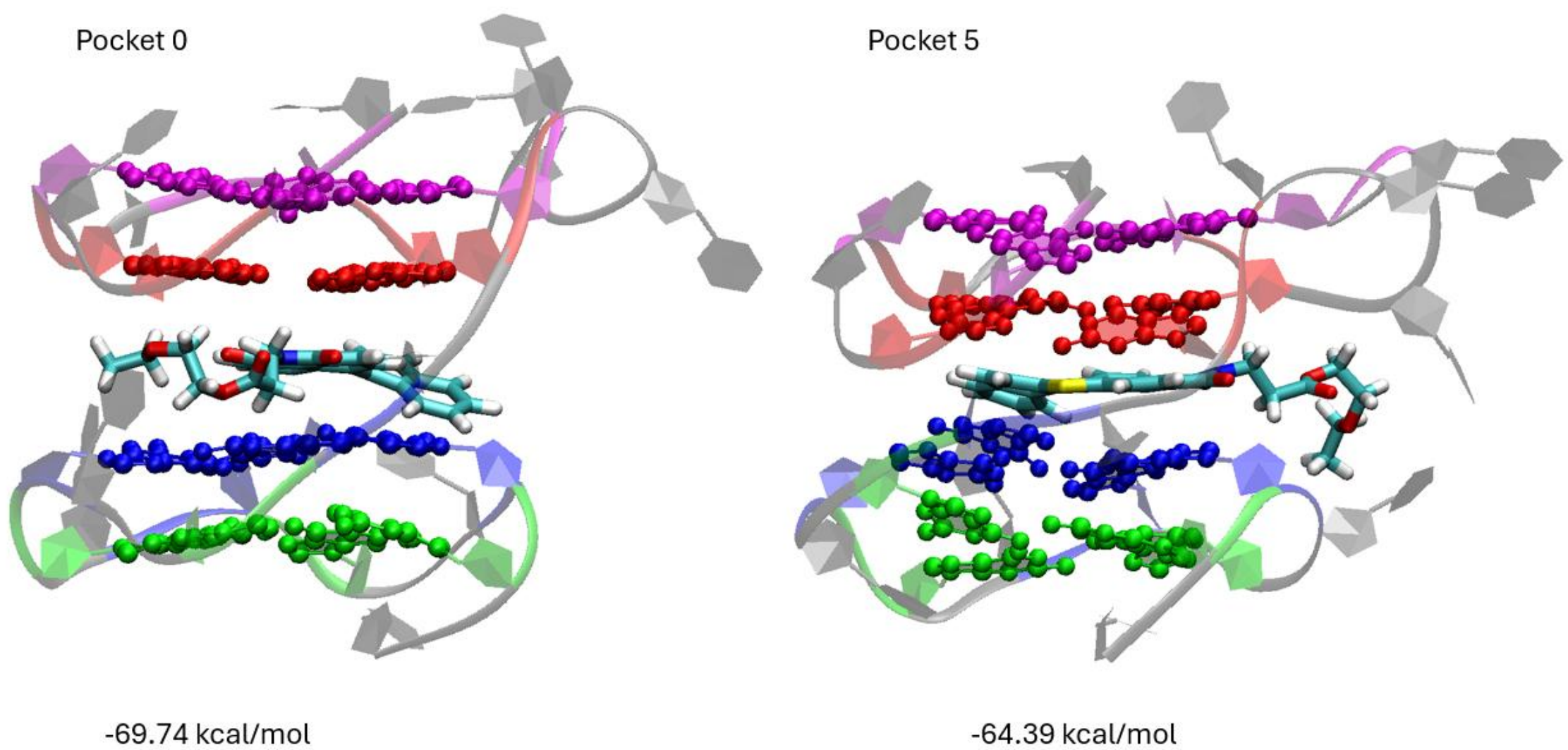

Figure S15. Representative structure 1000-2000 ns of the MD for pocket 0 (left) and 5 (right) in the central intercalative binding.

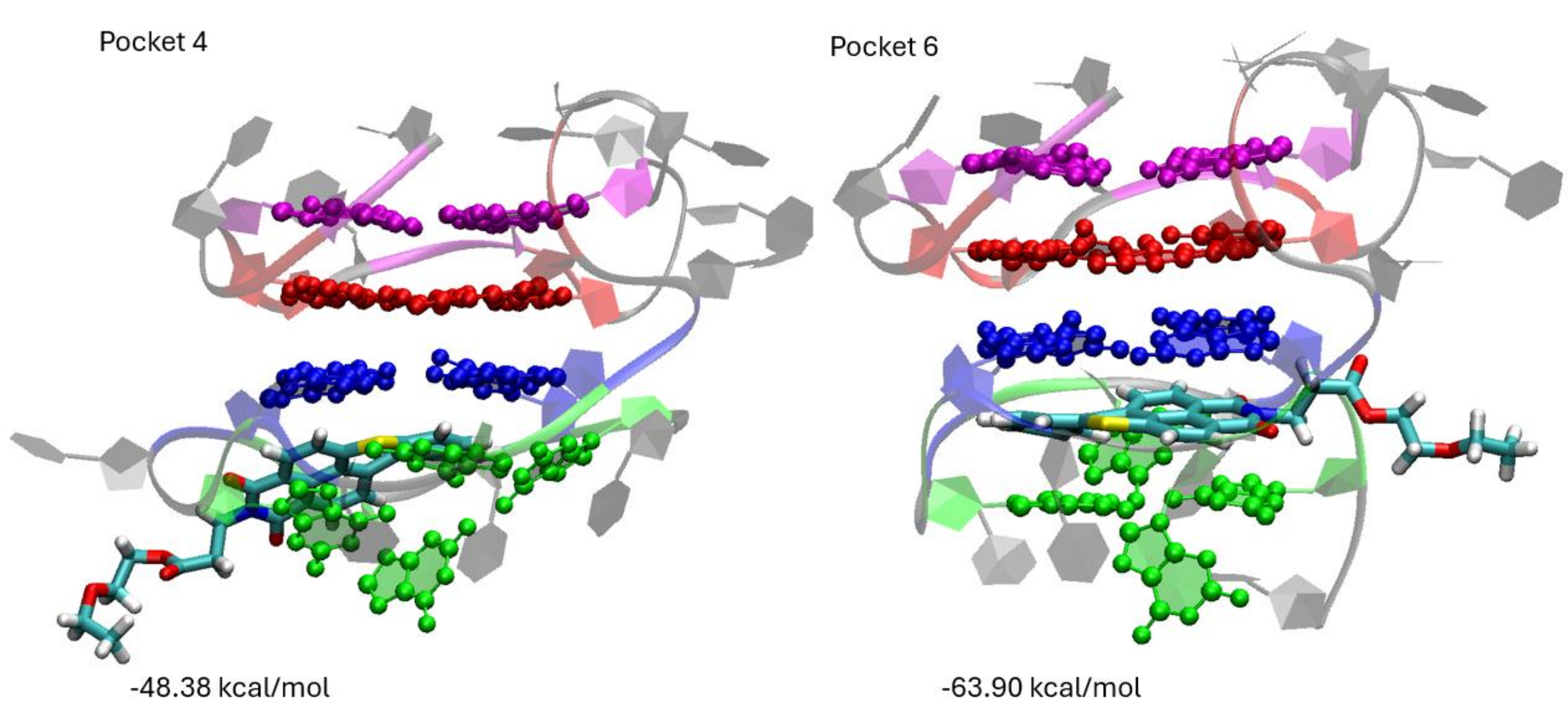

Figure S16. Representative structure 1000-2000 ns of the MD for pocket 4 (left) and 6 (right) in the *3′* intercalative binding.

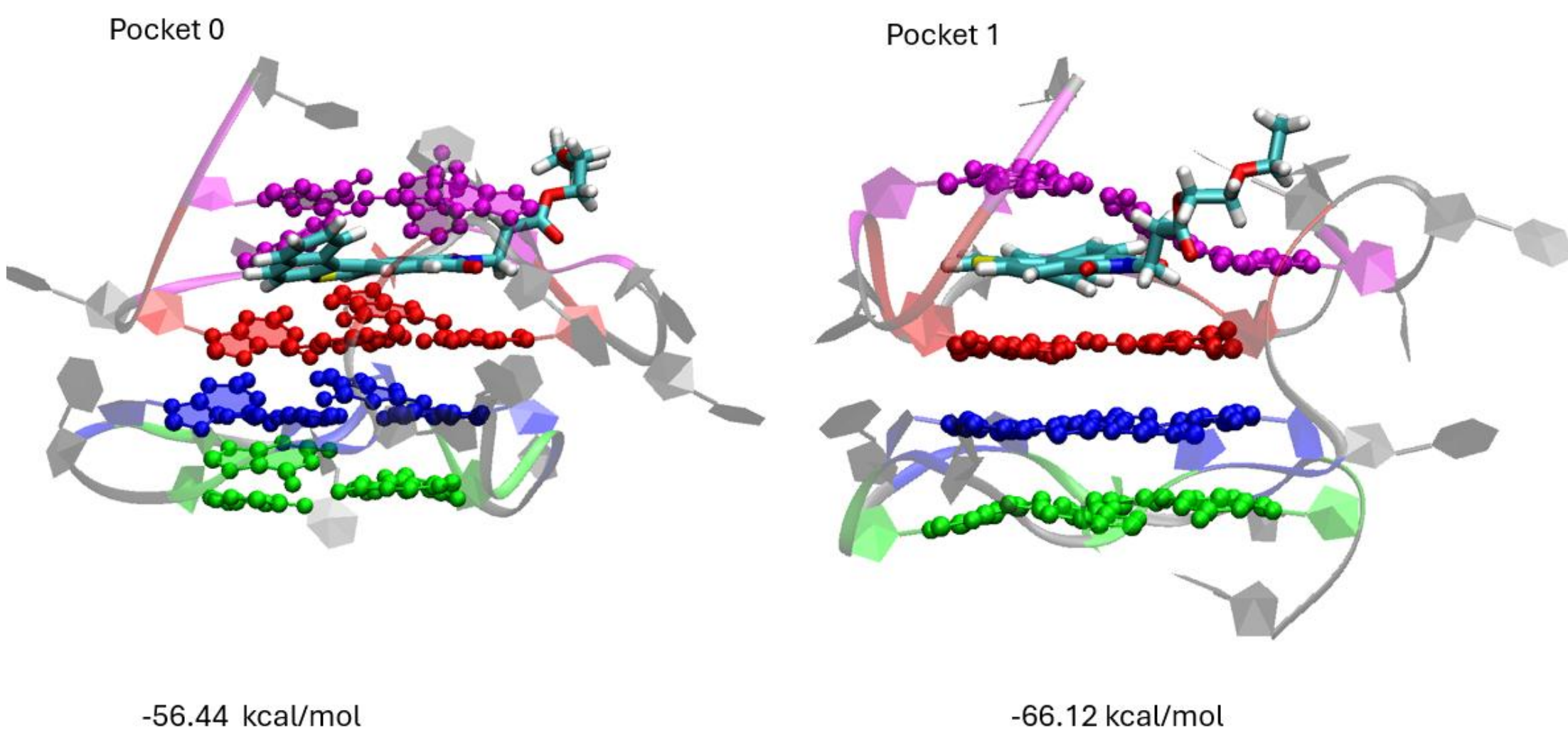

Figure S17. Representative structure 1000-2000 ns of the MD for pocket 0 (left) and 1 (right) in the *5′* intercalative binding.

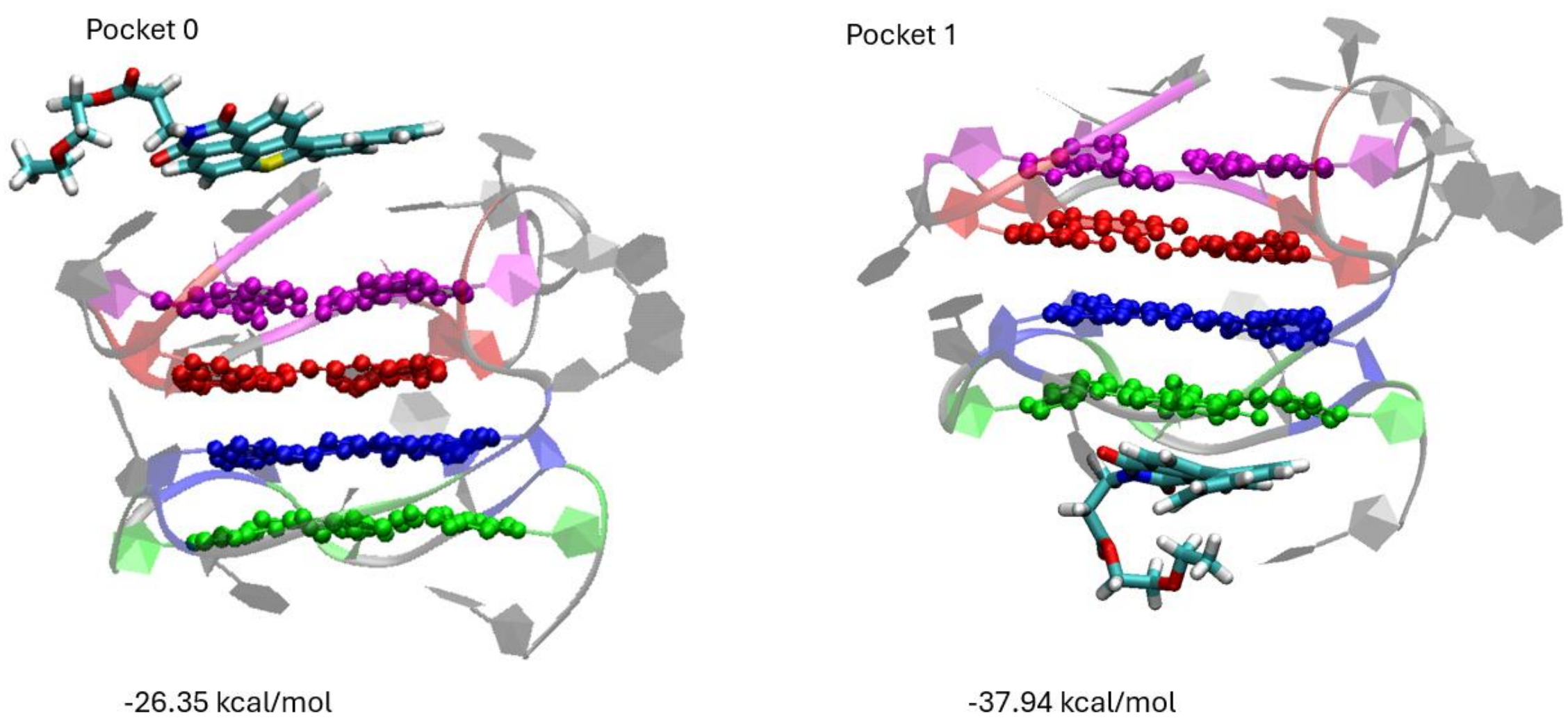

Figure S18. Representative structure 1000-2000 ns of the MD for pocket 0 (left) and 1 (right) in the external binding.

The last 1μs of the NPT production runs, with a 2-frame step (a total of 50000 frames), were employed to calculate the binding free energy of the resulting pockets by using the Molecular Mechanics Generalized Born Surface Area (MMGBSA) approach.[10] The calculated binding energies (Table S2, Figures S15–S18) of both central intercalative pockets and one intercalative *5′* and *3′* are very similar (in bold in Table S2), indicating that intercalative binding is possible in the three considered sites. In the case of external binding, binding close to the *3′* tetrad is preferred, although it seems less energetically favorable than intercalation.

Table S2. Energies in kcal/mol of the first and second MD simulated pockets for the intercalative and external binding.

	E First pocket (kcal/mol)	E Second pocket (kcal/mol)
Intercalated central	**-69.74**	**-64.39**
Intercalated *3′*	-48.38	**-63.90**
Intercalated *5′*	-56.44	**-66.12**
External	-26.35	-37.94

The MD simulations of the oxidized *AS1411* followed a similar procedure. However, 8-oxoG was described by GAFF2 instead of OL15[11], according to the standard antechamber parametrization of bound ligands. To obtain the ESP charges, 8-oxoG was optimized at the M05-2X[12]/6-31G* level of theory in implicit water solvation (IEFPCM) with Gaussian16. Then, the RESP charges were calculated at the Hartree-Fock/6-31G* level of theory with Gaussian16. Based on the experimental findings, we performed simulations with 8-oxoG mutations at G18, G21 and G24. Following the same minimization steps as before, the same NVT and NPT heating/equilibration steps were performed with no restrictions. Then a 2 μs NPT production was calculated. We ran simulations with and without the coordinated K^+ ions. Our results show the G4 remains stable during the simulations (Figure S19), although, at the end of the simulation without K^+, the structure of the *3′* tetrad starts to be disrupted, whereas the G4 still keeps its structure in the simulation with K^+ (Figures S20–S21).

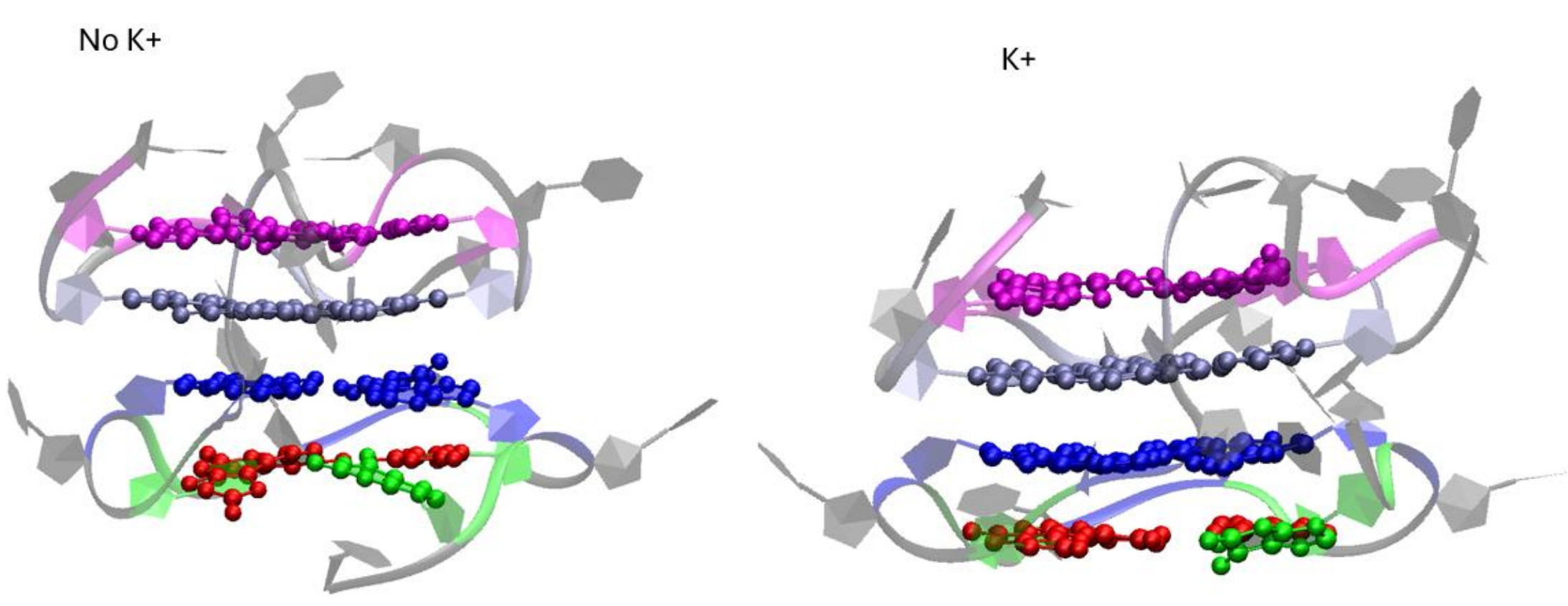

Figure S19. Representative structure 1000-2000 ns of the MD with 8-oxoG (red) at positions 18, 21 and 24 and no intercalated K^+ ions (left) and intercalated K^+ ions (right).

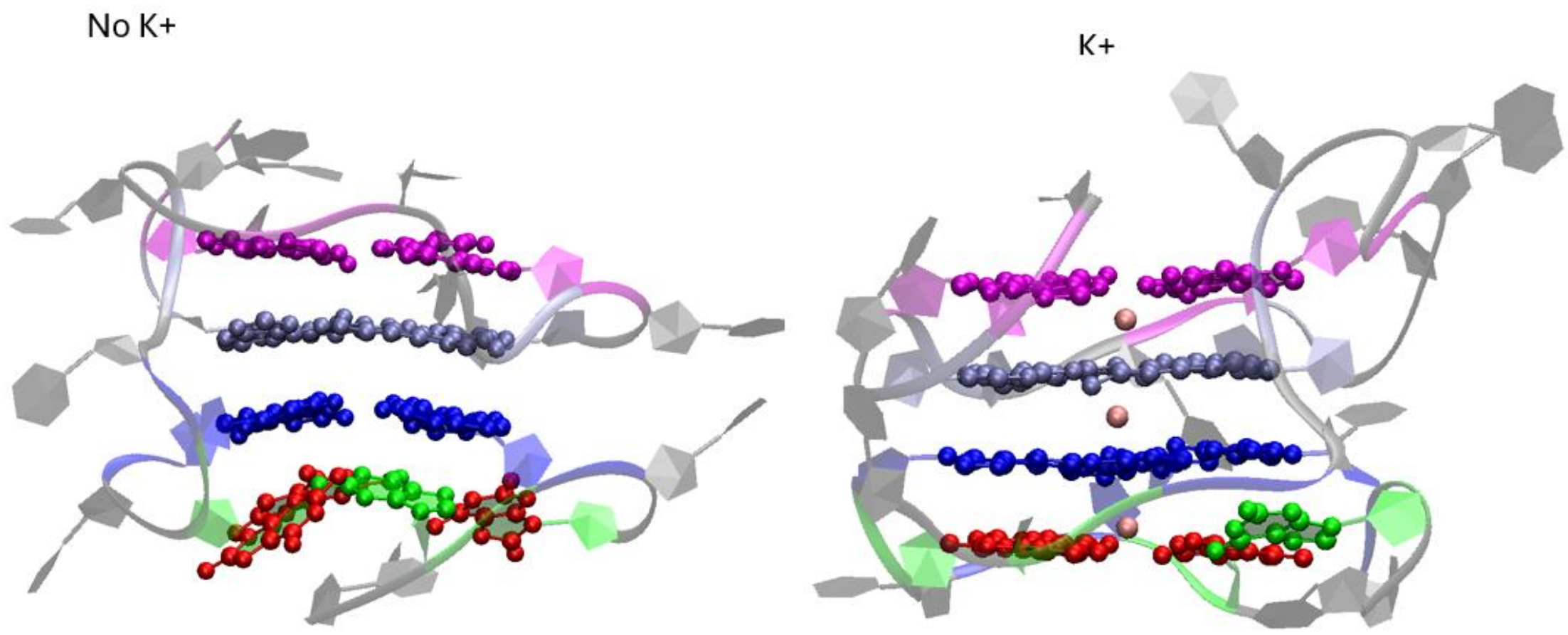

Figure S20. Last snapshot of the MD trajectory with 8-oxoG (red) at positions 18, 21 and 24 and no intercalated K^+ ions (left) and intercalated K^+ ions (right).

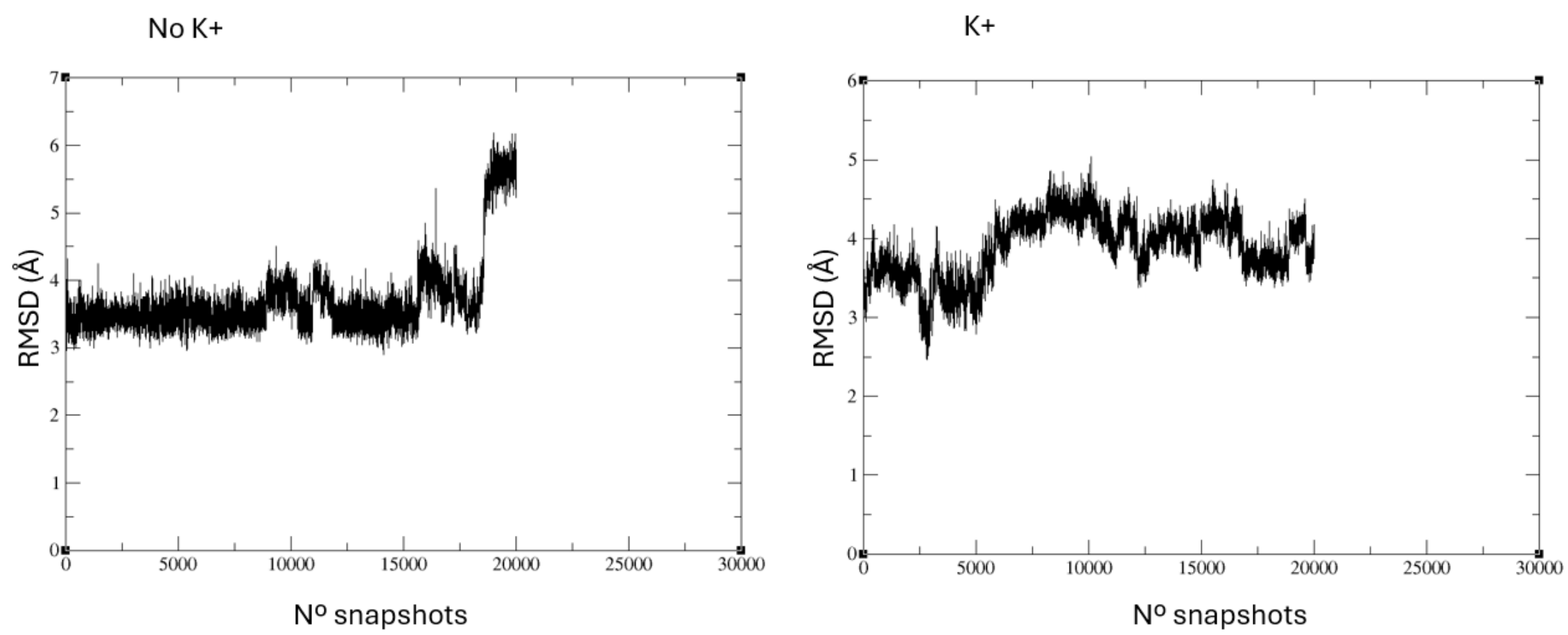

Figure S21. RMSD of the MD trajectory with 8-oxoG at positions 18, 21 and 24 and no intercalated K^+ ions (left) and intercalated K^+ ions (right).

The analysis of the trajectories included the calculation of the root mean square deviation (RMSD) to check the stability of the structures, and the dbscan clustering of the last 1 μs of the NPT productions with cpptraj. Moreover, VMD[13] was used to generate the computational figures in this work.

10.2. Quantum Mechanics calculations

QM Method

Density Functional Theory (DFT) and its time dependent (TD-DFT) version was used to study the different intercalation position/modes of **DBI-POE** to the aptamer, their absorption and circular dichroism properties, and for the most probable system its photophysics. In particular we select the M062X[14] functional combined with the 6-31G*[15] basis set and using an implicit model for water solvation (Polarizable Continuum Model, PCM[16, 17]). This method has been selected due to its good performance in similar studies.[1] Triplet state geometries were optimized resorting to the Tamm–Dancoff approximation (TDA)[18]. All the calculations were done with Gaussian 16 except for the Spin Orbit Coupling (SOC) terms that were computed using the pysoc[19] program.

Absorption and CD spectra were simulated by shifting the energies by 0.4 eV (necessary to match the experimental absorption maximum) and convoluting them with Gaussian functions of hwhm 0.3 eV.

QM Results

The selected representative structures of the MD were selected as our starting point for the QM calculations about the photophysics of the **DBI-POE** inside the aptamer. In detail, we considered both intercalated central pockets (rep0 and rep5 in the following), the second intercalated pocket at *3′* and the second intercalated pocket at *5′*.

First, we optimized their ground state minima that are depicted in Figure S22 (except for 8-oxoG were the CD was computed directly on top of the MD structure). QM agrees with MD being the rep0 more stable in ~2.5 kcal/mol compared to rep5. Unfortunately, the stability between the central, *3′* and *5′* intercalated clusters cannot be compared due to the different number of atoms.

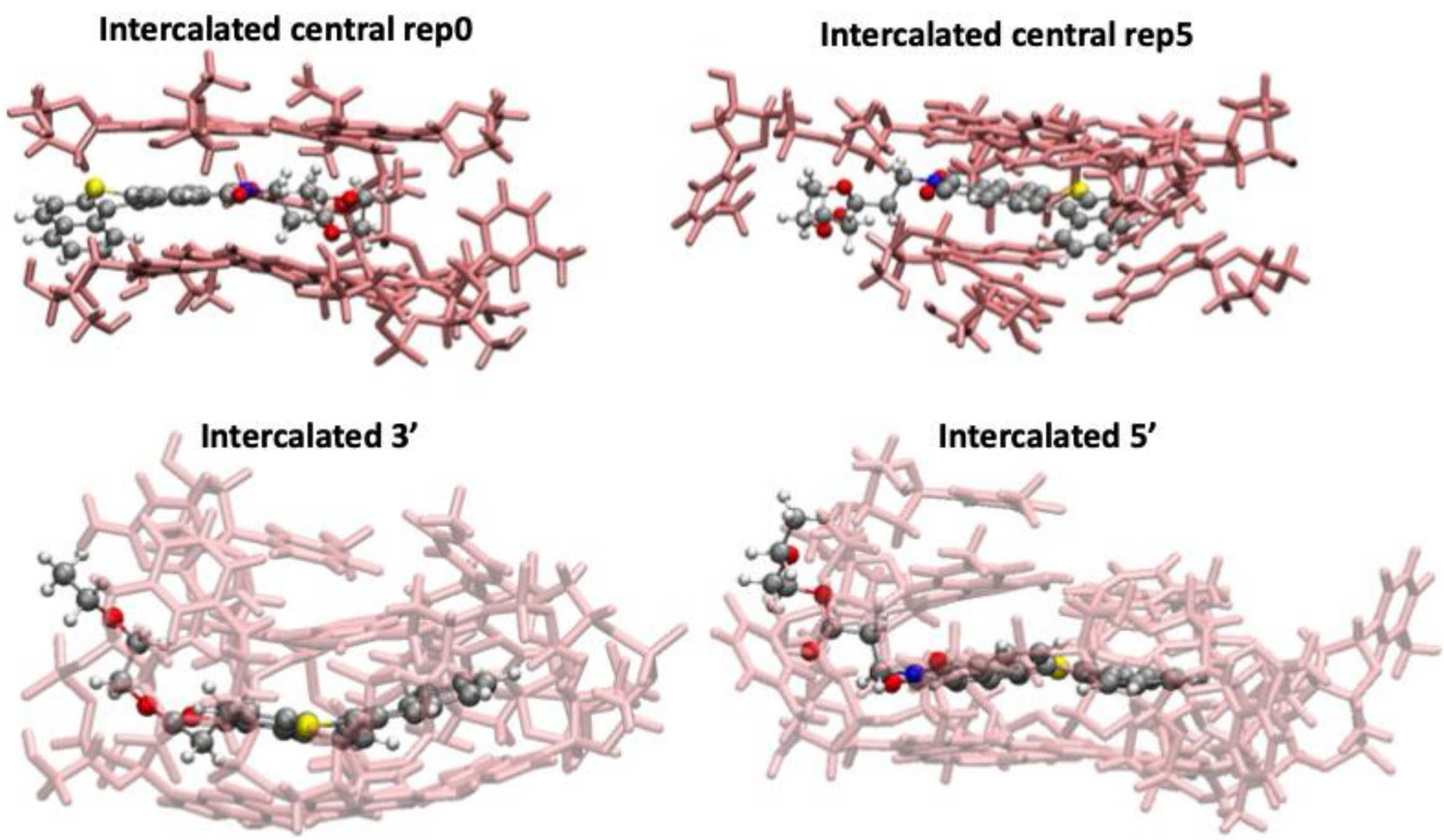

Figure S22. Ground state minima for all the systems computed at the QM level.

Then, their excited states (Table S3) were characterized at the Franck-Condon region and convolute them to simulate their absorption spectra (Figure S23A). As shown in the Figure, all the systems present an absorption maxima ~420-430 nm, mainly due to the bright S1 state (Table S4), except for rep0 system whose spectrum is significantly blue-shifted. We also see another incipient band at ~300 nm. In contrast their CD spectra (Figure S23B) are quite sensitive to the intercalation position and mode in the whole energy region: i) rep0 presents only a positive intense band ~260 nm, ii) rep5 and intercalated-*5´* are comparable following a slightly positive (~300nm), negative (~260 nm) and slightly positive (~240 nm) pattern and iii) intercalated *3´* with opposite trends, that is, negative (~280nm), positive (~250 nm) and slightly negative at the blue side.

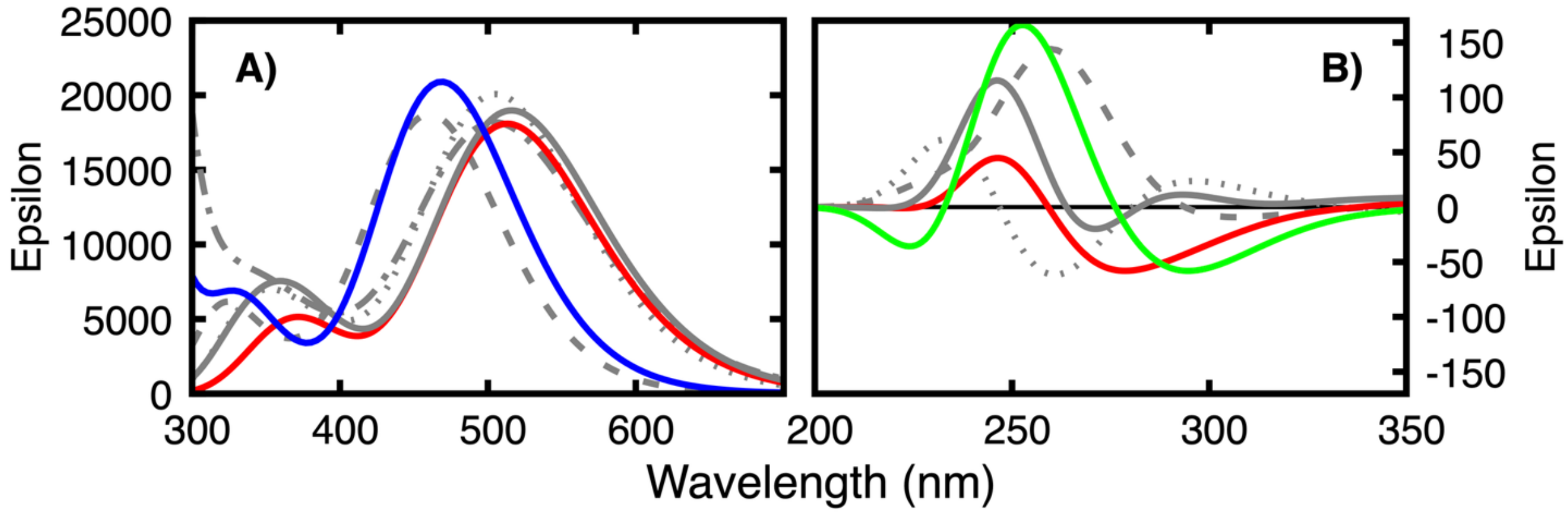

Figure S23. A) Absorption and **B)** CD spectra for all the systems computed at the QM level. Blue: **DBI-POE**, Red: intercalated *3′*, Solid gray: intercalated *5′*, Dashed gray: intercalated central rep0 and rep5. Green: CD spectrum for 8-oxoG.

Table S3. Excited state character, energies in eV (ΔE) and oscillator strength (f) for the lowest lying (below 4 eV) excited state of **DBI-POE** and **DBI-POE** intercalated at *3′* at the Franck-Condon region. When present, the amount of charge transfer (CT) character is specified in red in a.u.

	Character	ΔE	f		Character	ΔE	f
	**intercalated 3'**				**Monomer**		
S_1	ππ* DBI-POE	2.82	0.4017	S_1	ππ* DBI-POE	2.99	0.5934
S_2	ππ* DBI-POE + CT 0.2	3.49	0.0117	S_2	ππ* DBI-POE	3.68	0.0315
S_3	CT G1-> DBI-POE 0.6	3.66	0.0427	S_3	Inter-CT	4.14	0.1636
S_4	CT G4,5->DBI-POE 0.5	3.74	0.0436				
S_5	CT G4,6-> DBI-POE 0.8	3.79	0.0011				
S_6	CT Gs-> DBI-POE 0.7	3.91	0.0103				
S_7	CT G8-> DBI-POE 0.8	3.96	0.0052				
S_8	CT G3-> DBI-POE 0.8	3.98	0.0077				

Table S4. Initially optimized state from Franck-Condon region, excited state character, emission energies (and adiabatic in bold) in eV and oscillator strength (f) for the optimized minima of **DBI-POE** and **DBI-POE** intercalated at *3′*. When present, the amount of charge transfer (CT) character is specified in red in a.u.

	Character	ΔE	f		Character	ΔE	f
**intercalated 3'**				**Monomer**			
S_1	S_{1min} ππ* DBI-POE	2.41<br>**2.40**	0.6801	S_1	S_{1min} ππ*	2.45<br>**2.66**	0.7744
S_2	S_{2min} DBI-POE	3.09<br>**3.29**	0.0638	S_2	S_{2min}	3.09<br>**3.37**	0.1141
S_3	S_{3min} CT G1- DBI-POE 0.3	3.32<br>**3.28**	0.0778	S_3	S_{3min}	3.57<br>**3.87**	0.4350
S_4	S_{2min} CT G4- DBI-POE 0.7	2.58<br>**3.13**	0.0377				

Table S5. Energy difference (ΔE, in eV) and SOC terms (cm^{-1}) between the Sn and the different triplet states (Tn) at the position of singlet minima for **DBI-POE** intercalated at *3′*.

S_{1min}	ΔE	SOC	S_{2min}	ΔE	SOC	S_{2minCT}	ΔE	SOC
T1	-0.83	0.3	T1	-1.30	2.7	T1	-0.84	0.9
T2	0.11	1.3	T2	-0.68	0.3	T2	-0.09	1.1
T3	0.63	1.8	T3	0.11	0.7	T3	0.13	0.9
T4	0.76	1.6	T4	0.24	1.1	T4	0.61	1.5
T5	0.87	0.5	T5	0.39	1.2	T5	0.66	2.4
T6	0.93	1.8	T6	0.49	0.5	T6	0.76	1.0
T7	1.01	1.1	T7	0.50	0.5	T7	0.79	0.2
T8	1.07	0.5	T8	0.51	0.0	T8	0.85	1.2
T9	1.08	1.5	T9	0.54	0.0	T9	0.92	1.1
T10	1.12	1.5	T10	0.56	0.0	T10	0.94	0.9

Supplementary References

1. K. Saczuk, M. V. Cottini, M. Dudek, L. M. Mazur, D. P. Sánchez, L. López-Pacios, A. Kassem, K. Matczyszyn, J. J. Nogueira, C. Monnereau, L. Martínez-Fernández, J. Jamroskovic, C. Cabanetos and M. Deiana, Nanoscale Horizons, 2025, 10, 1660-1673.
2. J. Lyu, R. Shao, P. Y. Kwong Yung and S. J. Elsässer, Nucleic Acids Research, 2022, 50, e13-e13.
3. H. M. Berman, J. Westbrook, Z. Feng, G. Gilliland, T. N. Bhat, H. Weissig, I. N. Shindyalov and P. E. Bourne, Nucleic Acids Research, 2000, 28, 235-242.
4. N. Q. Do, W. J. Chung, Thi Hong A. Truong, B. Heddi and A. T. Phan, Nucleic Acids Research, 2017, 45, 7487-7493.
5. M. Dudek, L. López-Pacios, N. Sabouri, J. J. Nogueira, L. Martinez-Fernandez and M. Deiana, Angewandte Chemie International Edition, 2025, 64, e202413000.
6. M. Dudek, L. López-Pacios, N. Sabouri, J. J. Nogueira, L. Martinez-Fernandez and M. Deiana, The Journal of Physical Chemistry Letters, 2024, 15, 9757-9765.
7. W. J. Allen, T. E. Balius, S. Mukherjee, S. R. Brozell, D. T. Moustakas, P. T. Lang, D. A. Case, I. D. Kuntz and R. C. Rizzo, Journal of Computational Chemistry, 2015, 36, 1132-1156.
8. D. R. Roe and T. E. Cheatham, III, Journal of Chemical Theory and Computation, 2013, 9, 3084-3095.
9. J. Wang, R. M. Wolf, J. W. Caldwell, P. A. Kollman and D. A. Case, Journal of Computational Chemistry, 2004, 25, 1157-1174.
10. S. Genheden and U. Ryde, Expert Opinion on Drug Discovery, 2015, 10, 449-461.
11. R. Galindo-Murillo, J. C. Robertson, M. Zgarbová, J. Šponer, M. Otyepka, P. Jurečka and T. E. Cheatham, III, Journal of Chemical Theory and Computation, 2016, 12, 4114-4127.
12. Y. Zhao, N. E. Schultz and D. G. Truhlar, Journal of Chemical Theory and Computation, 2006, 2, 364-382.
13. W. Humphrey, A. Dalke and K. Schulten, Journal of Molecular Graphics, 1996, 14, 33-38.
14. Y. Zhao and D. G. Truhlar, Theoretical Chemistry Accounts, 2008, 120, 215-241.
15. P. C. Hariharan and J. A. Pople, Theoretica chimica acta, 1973, 28, 213-222.
16. J. Tomasi, B. Mennucci and R. Cammi, Chemical Reviews, 2005, 105, 2999-3094.
17. S. Miertuš, E. Scrocco and J. Tomasi, Chemical Physics, 1981, 55, 117-129.
18. S. Hirata and M. Head-Gordon, Chemical Physics Letters, 1999, 314, 291-299.
19. X. Gao, S. Bai, D. Fazzi, T. Niehaus, M. Barbatti and W. Thiel, Journal of Chemical Theory and Computation, 2017, 13, 515-524.
